# Macaque anterior cingulate cortex deactivation impairs performance and alters lateral prefrontal oscillatory activities in a rule-switching task

**DOI:** 10.1371/journal.pbio.3000045

**Published:** 2019-07-11

**Authors:** Liya Ma, Jason L. Chan, Kevin Johnston, Stephen G. Lomber, Stefan Everling

**Affiliations:** 1 Robarts Research Institute, University of Western Ontario, London, Ontario, Canada; 2 Graduate Program in Neuroscience, University of Western Ontario, London, Ontario, Canada; 3 Department of Physiology and Pharmacology, University of Western Ontario, London, Ontario, Canada; 4 Department of Psychology, University of Western Ontario, London, Ontario, Canada; 5 Department of Brain and Mind Institute, University of Western Ontario, London, Ontario, Canada; INSERM U846, Stem Cell and Brain Research Institute, FRANCE

## Abstract

In primates, both the dorsal anterior cingulate cortex (dACC) and the dorsolateral prefrontal cortex (dlPFC) are key regions of the frontoparietal cognitive control network. To study the role of the dACC and its communication with the dlPFC in cognitive control, we recorded local field potentials (LFPs) from the dlPFC before and during the reversible deactivation of the dACC, in macaque monkeys engaging in uncued switches between 2 stimulus-response rules, namely prosaccade and antisaccade. Cryogenic dACC deactivation impaired response accuracy during maintenance of—but not the initial switching to—the cognitively demanding antisaccade rule, which coincided with a reduction in task-related theta activity and the correct-error (C-E) difference in dlPFC beta-band power. During both rule switching and maintenance, dACC deactivation prolonged the animals’ reaction time and reduced task-related alpha power in the dlPFC. Our findings support a role of the dACC in prefrontal oscillatory activities that are involved the maintenance of a new, challenging task rule.

## Abstract 2

Reversible deactivation of the dorsal anterior cingulate cortex — an area of the cognitive control network — impairs rule maintenance but not rule switching per se, and disrupts task-related oscillatory activities in the dorsolateral prefrontal cortex — another area of the same network.

## Introduction

Survival in a dynamic environment requires cognitive control, which is the brain’s ability to guide actions using relevant information in a given context while suppressing irrelevant input and to flexibly adjust such guidance when the context changes. As part of the primate cognitive control network, the dorsolateral prefrontal cortex (dlPFC) and the dorsal anterior cingulate cortex (dACC) have been shown to co-activate in various cognitively demanding tasks [1–5]. Although the literature suggests that the dACC monitors and evaluates the costs and benefits of controlled actions [6] and environments [7] while the dlPFC allocates and regulates the control needed to execute the chosen action [8,9], their functions often overlap. Similar to the dACC, the dlPFC also encodes reward expectation [10–12] and prediction error [13]. Hence, it is likely that the 2 regions interact closely during cognitive control processes [14], sharing information concerning contexts, actions, outcomes, and their values. Thus, understanding the mechanisms through which the 2 areas communicate will shed light on the neural processes of cognitive control.

To study the role of the dACC in cognitive control processes such as behavioral flexibility and the inhibition of irrelevant task rules, we trained macaque monkeys to perform uncued switches between 2 stimulus-response rules—looking either toward (prosaccade) or away from (antisaccade) a peripheral visual stimulus. We hypothesized that dACC deactivation would impair the animals’ task performance, because it was found to encode rule information well before the onset of the peripheral target stimulus [15,16]. Additionally, given the dACC’s involvement in feedback processing [17–20] and prediction error [21,22], dACC deactivation may result in perseveration and a delay to the rule switch. Alternatively, the dACC was suggested to be critical for sustaining effective choices based on reward history [23,24], which predicted an impairment in maintaining performance on the new rule but not a delay in rule switching per se. Lastly, the dACC’s role in controlling cognitive effort [25–31] predicts a deficit in post-switch performance maintenance, especially when the new rule is more cognitively demanding than the previous one while the reward remains the same. In short, it is possible that dACC deactivation may affect either or both processes of rule switching and rule maintenance.

To study the interaction between the dlPFC and dACC during cognitive control processes, we recorded local field potentials (LFPs) from the dlPFC before and during the reversible deactivation of dACC while the monkeys were performing the task. We hypothesized that changes in oscillatory activities in the dlPFC during dACC deactivation will reveal how the 2 regions communicate to fulfill their roles in cognitive control. Low-frequency oscillations from the theta to beta range (4–30 Hz) have been suggested to coordinate neural activities across brain regions to serve cognitive functions. In both the lateral PFC and dACC, theta (4–8 Hz) activities have been implicated in attention and cognitive control [16,32–35]. In humans, frontal midline theta, which is partly generated from the dACC [36,37], was suggested to initiate cognitive control by entraining other brain areas [38]. Attenuation in frontal midline theta during cognitive control was found in patients with Parkinson disease [39,40], autism spectrum disorders [41], and schizophrenia [42]. Direct current stimulation restored theta-phase synchrony between the medial and lateral frontal cortex and improved cognitive performance in schizophrenia patients [43]. While less studied than theta, prefrontal beta oscillations (13–30 Hz) have been suggested to orchestrate cell assemblies that maintain information in short-term memory [44], predict reaction time [45,46], and reflect action outcome [47]. In between the theta and beta bands, alpha rhythms (9–12 Hz) may help inhibit attention to irrelevant information [45,48].

Here, we found that cryogenic dACC deactivation impaired the animals’ task performance in a manner consistent with an impairment in the maintenance of a cognitively demanding rule. This was manifested by a lower plateau in response accuracy but no delay in the implementation of the antisaccade rule after a switch from the prosaccade rule. Correlated with this performance impairment was a reduction in the absolute difference in dlPFC fixation-period beta activities between correct and error trials, particularly in antisaccades after the initial establishment of the rule. This reduced correct-error (C-E) difference likely contributed to behavioral impairment given that dlPFC alpha and low beta-band power (9–20 Hz) encoded the task rules in a performance-dependent manner. We also observed a reduction in task-related theta activity (6–8 Hz) specifically during the maintenance of the antisaccade rule. Additionally, we found increased saccadic reaction time (SRT) with both rules and across post-switch stages, which coincided with a reduction in task-related alpha (9–16 Hz) activities in the dlPFC. Together, our findings suggest a critical role of the dACC in dlPFC oscillatory activities associated with the maintenance of a cognitively challenging rule in a feedback-based rule-switching task.

## Results

### dACC deactivation impaired behavioral performance on the rule-switch task

**Fig 1:**
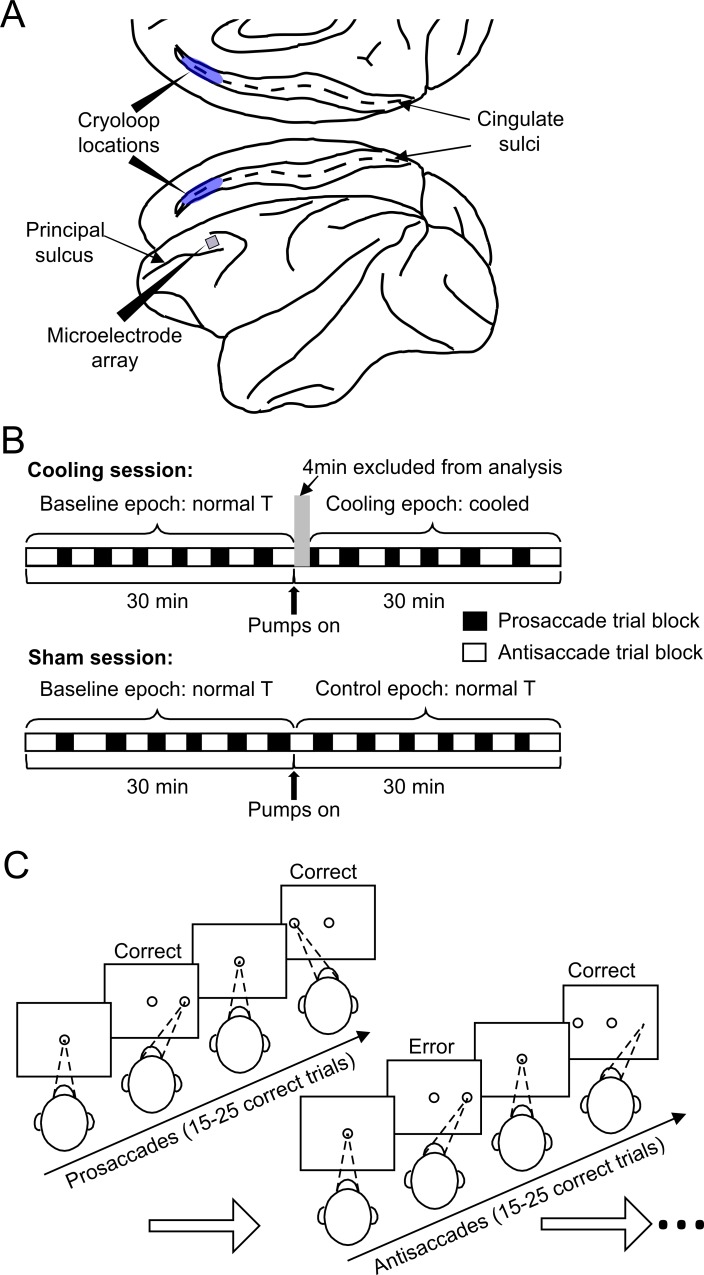
Schematics for the experimental setup, design, and behavioral paradigm. (A) The cryoloops were implanted bilaterally into the cingulate sulci (blue shades), and the microelectrode array was placed in the dlPFC of the left hemisphere. The posterior ends of both devices were placed at the same anterior-posterior coordinate as the posterior end of the principal sulcus. (B) At the beginning of a daily session, the animals were prepared similarly and performed the task for a 30-min baseline epoch before the pumps were turned on. In a cooling session, this was followed by a 30-min cooling epoch during which chilled methanol ran through the cryoloops, which was not the case for the 30-min control epoch in sham sessions. The first 4 min of the cooling epochs were excluded from analyses because the temperature of the tissue was transitioning to the target range. In each session, the animal completes 12–13 trial blocks under the prosaccade (empty rectangles) and the antisaccade (filled rectangles) rules, respectively. Cooling and sham sessions were conducted in a pseudorandom order. (C) To obtain a liquid reward, the animals were required to maintain fixation on the central white dot for a variable interval of 1.1 to 1.4 s, then make a saccade toward or away from the peripheral target according to the currently applicable rule. Once a block of 15 to 25 correct trials under one rule—prosaccade in this case—the rule was switched and had to be detected by trial and error, as indicated by the first trial of the antisaccade block in the schematic. dlPFC, dorsolateral prefrontal cortex.

To investigate the effects of cryogenic dACC (area 24c) deactivation on both behavioral performance and dlPFC (area 46/9d) activities (Fig 1A), we conducted 2 types of sessions. In “cooling sessions,” after a 30-min “baseline epoch” of behavioral and electrophysiological recordings, cold methanol was pumped through the implanted cryoloops to deactivate the dACC while the recording continued for another 30 min. Because the temperatures of the cryoloops took up to 4 min to drop below 20°C, the initial 4 min of the 30-min period were excluded from further analyses, and the rest of the 30 min constituted the “cooling epoch” (Fig 1B). Because in our design the cooling epoch took place after the baseline epoch and therefore was confounded with other variables that may affect behavior such as fatigue and reward satiation, we also conducted “sham sessions,” which alternated pseudorandomly with the cooling sessions. In sham sessions, the animals were prepared similarly and went through the first 30-min baseline epoch. For the following 30-min “control epoch,” the pumps were on but with no coolant running through the cryoloops. Thus, any difference in behavioral performance or neural activities between the baseline and control epochs in the sham sessions reflects the effect of epoch, whereas in the cooling sessions, such a difference reflects the combined effects of both epoch and dACC deactivation. Hence, a comparison between these differences reveals any effect specific to cooling per se.

**Fig 2:**
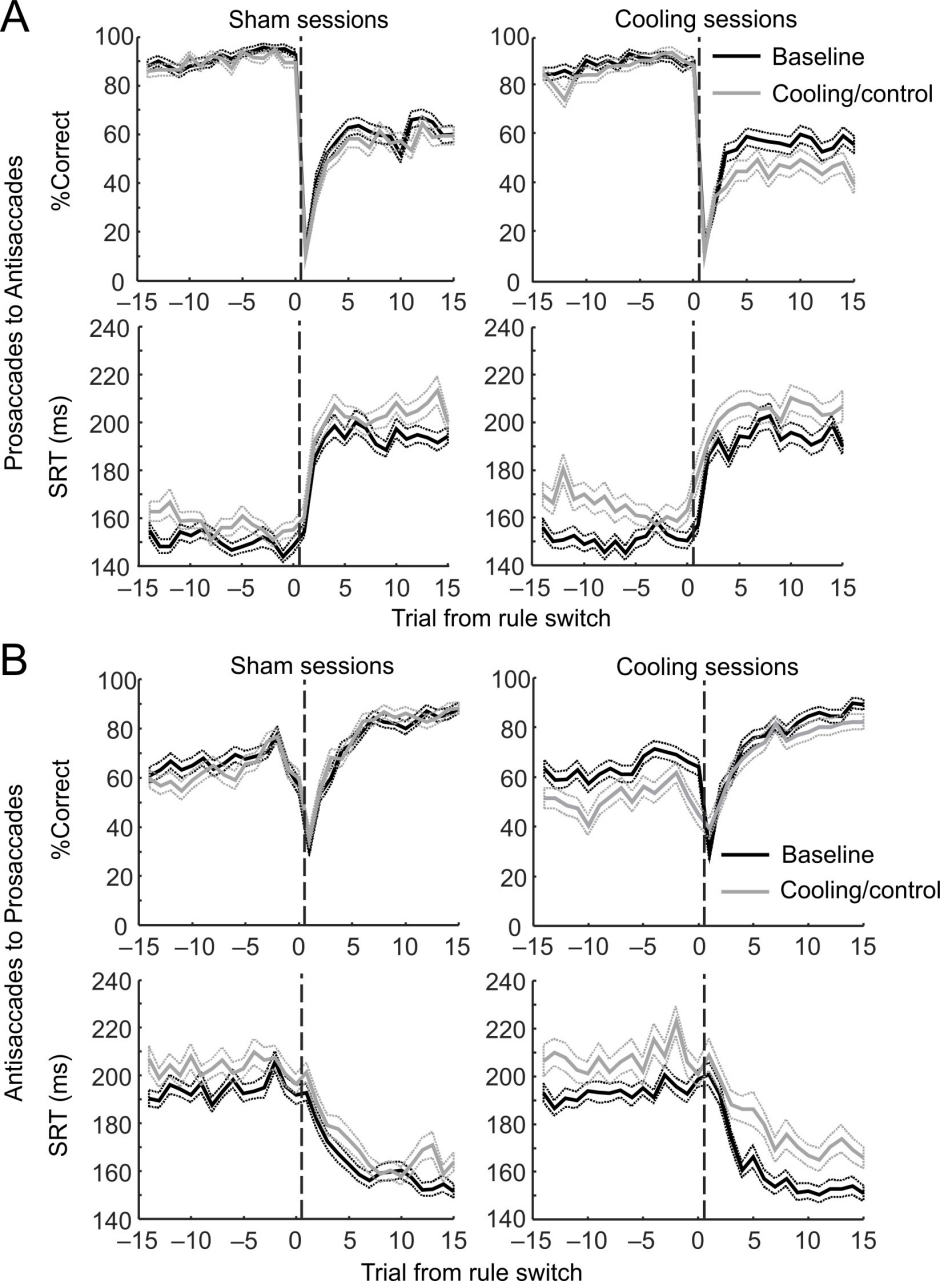
dACC deactivation impaired behavioral performance in antisaccade trials and increased the SRTs under both rules. See S1 Table and S2 Table for complete lists of test statistics, *p*-values, effect sizes, and statistical power. (A) Switches from the prosaccade to the antisaccade rule. Top panels: in both sham (left) and cooling (right) sessions, percentages of correct trials—averaged across all trial blocks completed—dropped sharply at the first trial after the rule switches but rose quickly (the Early stage) to a plateau at approximately the fifth trial post switch. In sham sessions (top left panel), the performance remained unchanged from the baseline (black curve) to the control epoch (gray curve) on either the pre-switch prosaccade trials (to the left of the dashed line) or the post-switch antisaccade trials (to the right of the dashed line). Cooling impaired the animals’ performance in the post-switch antisaccade trials (top right panel, gray versus black curves, to the right of the dashed line) but not in the pre-switch prosaccade trials (to the left of the dashed line). Bottom panels: cooling significantly increased the SRTs of correct responses in both the pre-switch prosaccade responses and the post-switch antisaccade responses (gray versus black curves, bottom right panel) and more strongly than epoch alone (gray versus black curves, bottom left panel). (B) Switches from the antisaccade to the prosaccade rule. Top panels: similar to the top panels in (A), the animals’ performance went through the Early post-switch stage in which the performance rose sharply, and the Stable stage in which the performance was maintained or improved at a much slower pace. Cooling impaired the animals’ performance in the pre-switch antisaccade trials (top right panel, gray versus black curves, to the left of the dashed line) but not in the post-switch prosaccade trials (to the right of the dashed line). No such effect was found for either rules between the baseline and the control epochs (top left panel). Bottom panels: cooling (bottom right panel) but not epoch per se (bottom left panel) significantly increased the SRTs in both the pre-switch antisaccade responses and the post-switch prosaccade responses. The dotted lines indicate standard error of the mean. Data associated with this figure can be found at 10.6084/m9.figshare.8236589. dACC, dorsal anterior cingulate cortex; SRT, saccadic reaction time.

**Fig 3:**
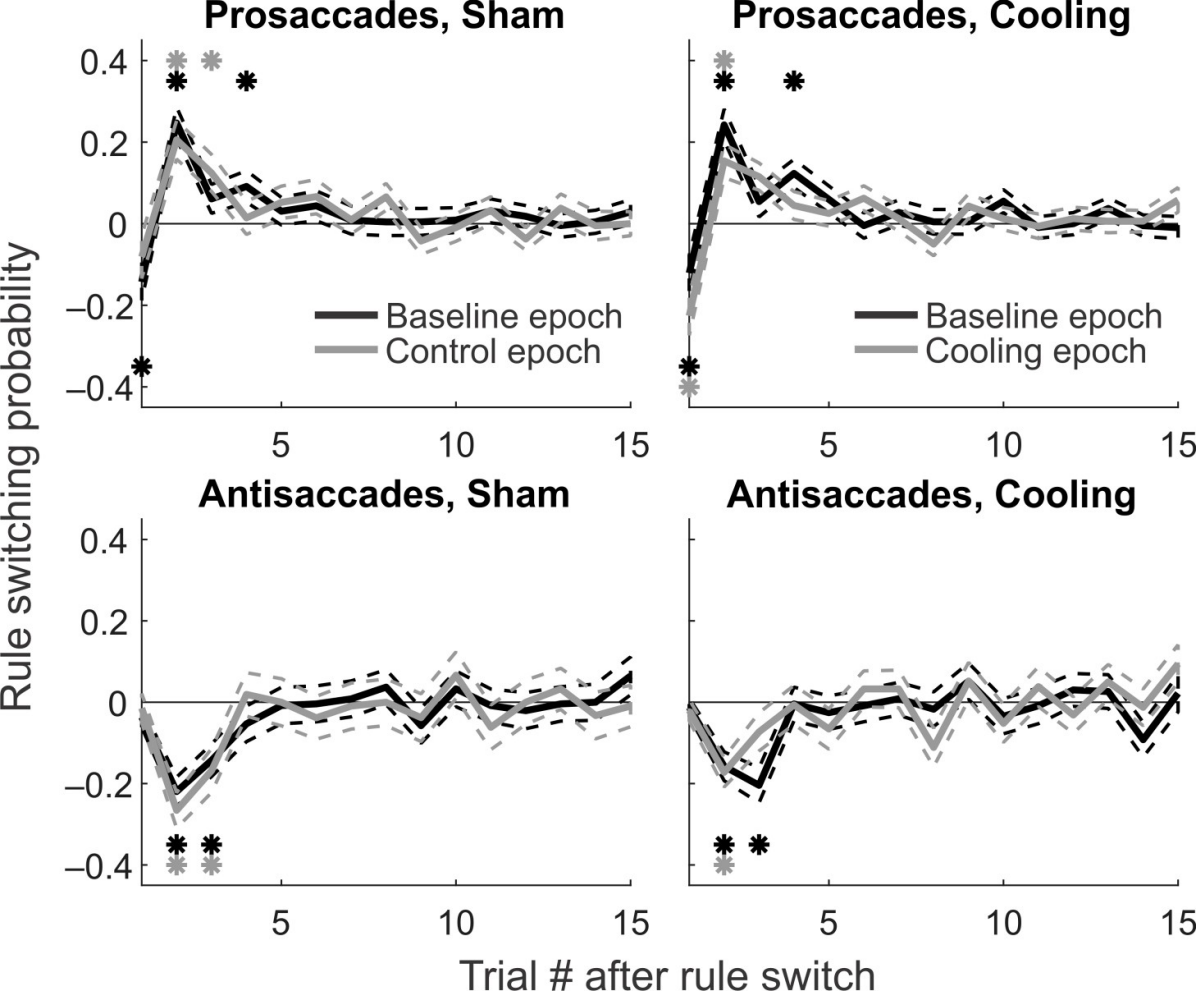
**Signed rule-switching probability under either rule (top versus bottom row) in cooling or sham sessions (left versus right column) during baseline (black curves) and control/cooling epochs (gray curves).** Asterisks indicate significantly positive or negative switching probability in the curve with the same color. Positive switching probability indicates that a switch from antisaccade to prosaccade rule often occurred from the previous trial to the current one. Negative switching probability indicates that a switch in the opposite direction often took place. Top left: in sham sessions, switching from the antisaccade to the prosaccade rule usually took place between the second and fourth trial post switch. Toward the end of an antisaccade trial block, the animals often tested the prosaccade rule and then switched back to antisaccades, as reflected in the negative switching probability in the first post-switch trial. Top right: similar pattern was observed in cooling sessions. Cooling did not disrupt or delay the switch from antisaccades to prosaccades. Bottom left: in sham sessions, switching from the prosaccade to the antisaccade rule usually took place in the second or third trial post switch in both the baseline (black) and control (gray) epochs. Bottom right: cooling did not disrupt or delay the switch from prosaccades to antisaccades. Thus, significant rule switches were completed within the first 4 trials of a new block of either prosaccade or antisaccade trials, which we termed the Early stage. Data associated with this figure can be found at 10.6084/m9.figshare.8236589.

Throughout both cooling and sham sessions, the monkeys performed the rule-switch task. In the case illustrated in Fig 1C, the animal started with the prosaccade rule. Each trial started with the onset of a white fixation dot at the center of the screen. The animal had to acquire and maintain fixation for a random interval ranging from 1.1 s to 1.4 s, until the onset of a peripheral stimulus, to which a prosaccade was required for the delivery of water reward. After a random number of between 15 and 25 correct trials, the task rule was switched. In this case, when the peripheral stimulus appeared, the animal was required to generate an antisaccade away from it to the mirror location. The post-switch portions of the performance plots displayed 2 phases (Fig 2, curves to the right of the dashed line): the animals typically detected the rule switch through trial and error within approximately the first 4 trials post switch, which we tentatively labeled as the Early stage; after this, they came to adopt the new rule and reached the Stable stage of performance, for which we included the 8 trials following the Early stage (Fig 2A, upper panels). While this grouping of trials was based on visual inspection of Fig 2, in the next analysis (Fig 3), we examined whether this grouping was supported by the signed rule-switch probability for each serial position in a trial block. For the analyses of SRTs, we only included correct trials because the same SRT on different error trials may be caused by diverse erroneous processes. The SRTs also went through the Early and Stable stages as task rules switched (Fig 2A, lower panels). Because antisaccades generally have longer SRTs than prosaccades, this switch led to an increase in SRTs in the Early stage that was sustained throughout the block of antisaccade trials (Fig 2A, lower panels). At the end of the block, the rule was switched again; after this, the animals appeared to go through the same 2 stages in performance (Fig 2B, upper panels). Switching from antisaccade to prosaccade (A→P) involved a decrease in SRTs (*F*_1,763_ = 959.4, *p <* 4.9 × 10^−324^; Fig 2B, bottom panels). Each type of switch—prosaccade to antisaccade (P→A) and vice versa—was repeated on average 12 to 13 times per session (Fig 1B). To avoid redundancy in testing, we will focus on the A→P switches. It should be noted that these switches involved more than simple reversal learning, since additional cognitive effort was required on antisaccade trials to suppress the prepotent prosaccade response. Hence, the ability to switch between rules with different difficulty required flexibility in the deployment of cognitive resources.

We examined the effect of epoch and cooling on task performance and averaged SRT using repeated-measures ANOVA on the 15-trial blocks before and after each A→P switch, with the “switch” as the within-subject variable (pre versus post, which coincided with the rule: antisaccade versus prosaccade) and the epoch (baseline versus cooling/control) and session type (cooling versus sham) as categorical factors (for a complete list of test statistics, *p*-values, effect sizes, and statistical power see S1 Table). In these tests, each 15-trial block contributed a single percentage of correct responses or a single averaged SRT. Because each cooling session contained a baseline epoch before cooling onset, a main effect of session type would not be necessary to show an effect of dACC deactivation. What we mainly looked for was an interaction between epoch and session type: we found no difference between the control epoch and the baseline epoch in sham sessions, compared with a significant difference between the cooling epoch and the baseline epoch in cooling sessions; or, if there was an effect of epoch in sham sessions, this effect was stronger or was in the opposite direction in cooling sessions. In percentage of correct responses, we found an interactive effect of session type and epoch (*F*_1,764_ = 18.0, *p =* 2.5 × 10^−5^): performance deteriorated from baseline to cooling epochs in cooling sessions (post hoc Tukey’s test: *p =* 7.7 × 10^−6^) but not from baseline to control epochs in sham sessions (*p =* 0.86) (S1 Table, Fig 2B, upper panels). This effect was explained by a decrease in performance in pre-switch antisaccade trials (*p =* 3.2 × 10^−5^) but not post-switch prosaccade trials (*p =* 0.27) in cooling sessions. In the SRTs, we also found an interactive effect of epoch and session type (*F*_1,763_ = 4.03, *p =* 0.045). SRTs increased from baseline in both pre-switch antisaccade trials (post hoc Tukey’s test: *p =* 0.0069) and post-switch prosaccade trials (*p =* 3.4 × 10^−5^) in cooling but not sham sessions (*p =* 0.063 and 0.12 respectively; S2B Table, Fig 2B, bottom panels). Hence, dACC deactivation impaired the animals’ response accuracy under the more challenging antisaccade rule and increased their reaction times across both task rules. In the line plots, it appears that cooling affected the Stable stage more than the Early stage after the task switches, especially in the antisaccade trials (Fig 2A and 2B, upper right panels), compared with the controls (upper left panels).

So far, our definitions for the putative Early and Stable stages were based on visual inspection of Fig 2. In search for a more objective and quantitative criterion for post-switch stages, we calculated the signed rule-switch probability. This was done by firstly coding each prosaccade trial as 1 and antisaccade trial as 0; then, the signed rule switch was calculated by subtracting the code of the previous trial from that of the current one. For instance, the signed rule switch was 1 for a prosaccade trial if it followed an antisaccade trial; it would be −1 for an antisaccade following a prosaccade trial. If a trial followed the same rule as the trial before, then the signed rule switch would be 0. At each post-switch serial position (e.g., first or second trial post switch), we averaged the signed rule switch across all trial blocks to obtain the signed rule-switch probability (Fig 3). In prosaccade trials, up to the first 4 trials post switch demonstrated a significant probability for rule switch in both sham (left panels) and cooling sessions (right panels), during both baseline (black curves) and control/cooling epochs (gray curves; black and gray asterisks indicate family-wise false discovery-corrected *p* < 0.05 in one-sample *t* test against 0) [49,50]. On the second through fourth trials, the signed probability was positive, indicating that the animals frequently switched to the correct prosaccade rule. On the first trial, the sign was negative, as the animals tended to test the prosaccade rule towards the end of the previous antisaccade trial block—potentially as an attempt to save effort—and switched back to the antisaccade rule upon receiving negative feedback, unknowingly on the first trial of a new prosaccade block. They would then receive no reward following the antisaccade, thereby realizing the switch, demonstrated as a positive rule switch on the second trials. Similar shifts towards the new rule were observed on the second and third trials in antisaccade trial blocks, but not on the first trials because the animals could not have noticed the shift in rule before the first feedback (Fig 3, lower panels, black and gray asterisks).

For prosaccades, from baseline to control epoch, the last trial with significant rule-switch probability moved from the fourth to the third trial post switch (Fig 3, upper left panel, black and gray asterisks), which may demonstrate a within-session improvement in the animals’ rule-switch efficiency. From baseline to cooling epoch (Fig 3, upper right panel), this improvement appeared to be enhanced, with the last significant switch moving from the fourth to the second post-switch trial. Similar improvement was observed in antisaccade trials (black versus gray asterisks, Fig 3, lower panels). Hence, dACC cooling did not impair the animals’ ability to switch between rules. These results also demonstrated that significant rule switches were completed within the first 4 trials of a new block of either prosaccade or antisaccade trials, which supported an operative definition of these trials as the Early stage. To compare with this Early stage, we defined the fifth to the 12th trials post switch as the Stable stage, during which no significant unidirectional rule switch took place (Fig 3, all panels). Note that the lack of difference in antisaccade rule-switching probability before and during cooling (Fig 3, lower right panel) is not at conflict with results shown in Fig 2. Whereas Fig 2 demonstrates the probability of following a rule, Fig 3 illustrates the averaged probability of switching from or to a rule. The near-zero average indicates equal probabilities in both committing an error after a correct response and making a correct response after an error, both before and during dACC deactivation. While the lower plateau in Fig 2 indicates that the animals were more likely to make an error after responding correctly under the new rule during dACC cooling, Fig 3 suggests that they were equally likely to respond correctly after an error. Given that our task was uncued and was feedback based, the outcome of a given trial may affect the animal’s choice on the next trial. Additionally, if the animals had a side bias or adopted an incorrect strategy, e.g., always responding to one direction, the saccadic direction may also affect their action or accuracy on the following trial. We tested these possibilities using binomial logistic regression models. While the response accuracy on a trial affected the accuracy on the following trial in some cases—as expected for a feedback-based task, there was no evidence that the animals had a side bias or used an incorrect “direction strategy,” even when the rule was switched (see S1 Text).

Although correct responses appeared to be guided by task rules, for error responses—especially those under the antisaccade rule—it remained unclear whether the animals failed to maintain the rule or lost control over the impulse of looking towards the stimulus with dACC deactivation. Because impulsive errors usually have shorter reaction times, a decrease in SRTs during direction errors would suggest a loss of impulse control. We conducted a two-way ANOVA on the effects of epoch and session type on the SRTs of error responses in antisaccade trials. Overall, SRTs increased with epoch (*F*_1,4879_ = 36.8, *p =* 1.4 × 10^−9^), which did not interact with session type (*F*_1,4879_ = 0.064, *p =* 0.80). That is, SRTs of direction errors in antisaccade trials (i.e., prosaccades made under the antisaccade rule) increased with epoch similarly in cooling (post hoc Tukey’s test, *p =* 0.0002) and sham sessions (*p =* 5.7 × 10^−5^). This increase in SRTs suggested that the animals did not become more impulsive with epoch, which captures the combined effect of time and time-related factors such as satiation and fatigue. The lack of epoch × session type interaction suggested that dACC deactivation did not have a slowing effect on the SRTs of errors beyond the effect of epoch. That is, these SRTs increased to the same extent in both sessions (sham: 167.7 ± 60.8 ms to 178.1 ± 62.9 ms; cooling: 168.7 ± 58.6 ms to 180.0 ± 67.3 ms). Thus, neither time-related factors nor dACC deactivation weakened impulse control in the animals. Note that this finding does not rule out the possibility that under dACC deactivation, the animals were unable to use the rule to guide their behavior despite their knowledge of the current rule, due to reasons other than a loss of impulse control. For instance, dACC deactivation may have reduced their ability to raise or apply cognitive efforts when preparing for antisaccades.

For a thorough comparison, we plotted the distributions of SRTs of correct and error trials in different epochs and session types (S2 Fig) under both the antisaccade (S2A Fig) and prosaccade trials (S2B Fig). Given the large sample size, the difference in the 2 distributions visualized in each panel reached significance by Kolmogorov-Smirnov test (KS statistic from 0.054 to 0.25, *p*-value from 1.2 × 10^−70^ to 1.9 × 10^−5^), with the SRTs consistently greater in the control/cooling epochs than in the baseline epochs in all cases. Although the direction errors under the antisaccade rule—i.e., prosaccades—in cooling epochs had shorter SRTs (mean ± SD: 179.7 ± 60.1 ms) than correct antisaccades (232.1 ± 53.9 ms; lower versus upper right panels, S2A Fig), they were still not as fast as correct prosaccades (154.0 ± 50.2 ms; S2B Fig, upper right panel). Their SRTs were also very similar to the same type of errors during the control epochs (179.7 ± 60.1 ms versus 180.6 ± 62.3 ms). Overall, we found no evidence that either time and time-related factors or dACC deactivation increased impulsivity in the animals; instead, they prolonged the SRTs regardless of the response accuracy.

In short, while the animals’ performance was impaired by dACC deactivation, this was not associated with the use of an alternative, incorrect strategy (see S1 Text) or an increase in impulsive errors. Instead, it was likely due to a weakened representation of the current rule or the ability to use the rule to guide behavior.

### dACC deactivation specifically affected behavioral performance during rule maintenance

**Fig 4:**
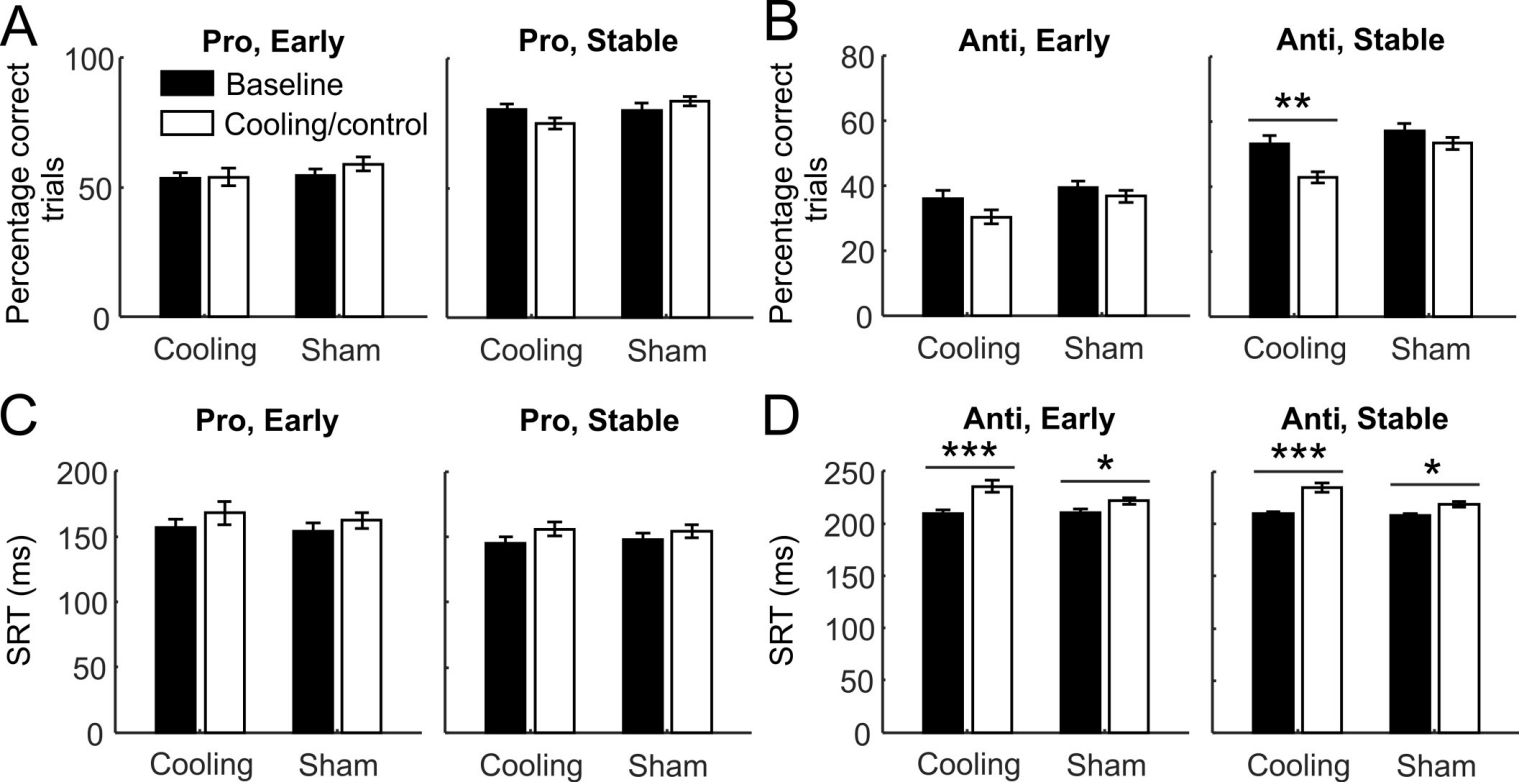
The impact of dACC deactivation on behavioral performance depended on the post-switch stage and the rule. Behavioral performance at 2 different post-switch stages. The Early stage included the first to the fourth trial post switch, and the Stable stage included the fifth to the 12th trial. See S3 and S4 Tables for complete sets of result from statistical tests. (A) Performance as measured by the percentage of correct trials did not change from the baseline to the cooling or control epoch in either the Early (left panel) or the Stable stage (right panel). (B) Cooling (left bars) but not epoch per se (right bars) significantly impaired the animals’ performance at the Stable stage (right panel) but not the Early stage (left panel). (C) Cooling did not affect the SRTs of correct responses in prosaccade trials in either stage alone, but when considered together, it increased the SRTs more strongly in cooling than in control epoch compared with the baseline epochs. (D) There was a cooling × epoch interaction: while the SRTs in both the Early (left panel) and the Stable stages (right panel) increased from the baseline to the control epochs (right bars), they increased more strongly from the baseline to the cooling epochs (left bars). The error bars indicate standard error of the mean. Asterisks indicate significant difference by post hoc Tukey’s test between adjacent bars below the horizontal lines. Data associated with this figure can be found at 10.6084/m9.figshare.8236589. *p < 0.05 **p < 0.001 ***p < 0.0005. dACC, dorsal anterior cingulate cortex; SRT, saccadic reaction time.

Because each rule switch elicited a two-stage adaptation to the new rule (Figs 2 and 3), we now included post-switch stage as an additional factor in our behavioral and neural analyses. Because each block of consecutive Early-stage trials only contained 4 trials, we pooled all Early-stage trials from a given experimental session to produce a single percentage of correct responses and an averaged SRT. The same was done for Stable-stage trials. We performed a repeated-measures ANOVA for prosaccade and antisaccade trials, respectively, with post-switch stage and epoch as within-subject variables and cooling as a categorical factor. Any effect of cooling and not epoch alone should take the form of an interaction between epoch and session type: LFP power during the baseline and cooling epochs of cooling sessions would be expected to be more different than those between the 2 epochs of sham sessions. In prosaccade trials, neither epoch (*F*_1,58_ = 0.186, *p =* 0.67) nor session type (*F*_1,58_ = 1.76, *p =* 0.19) affected response accuracy, although there was a trend toward an interaction between epoch and session type (*F*_1,58_ = 3.34, *p =* 0.073; S3A Table, Fig 4A). By contrast, in antisaccade trials, cooling impaired the animals’ performance (main effect of session type: *F*_1,58_ = 7.23, *p =* 0.0094, of epoch: *F*_1,58_ = 12.06, *p =* 0.00098), specifically in the Stable (post hoc Tukey’s test: *p =* 0.00062) but not the Early stage post switch (*p =* 0.21; S3B Table, Fig 4B, left bars in each panel). In sham sessions, performance on antisaccade trials did not change between the baseline and control epoch (*p =* 0.94 and 0.71 in Early and Stable stages, respectively, Fig 4B, right bars in each panel). Thus, dACC deactivation impaired the animals’ ability to achieve a high level of stabilized performance under the antisaccade rule.

For correct SRTs, the same analyses were conducted. Unlike the changes in performance, on prosaccade trials we found a main effect of epoch (repeated-measures ANOVA, *F*_1,58_ = 23.14, *p =* 1.1 × 10^−5^) but not session type (*F*_1,58_ = 0.037, *p =* 0.85) or epoch × session type interaction (*F*_1,58_ = 1.11, *p =* 0.30). The SRTs increased in both cooling (post hoc Tukey’s test: *p =* 0.00076) and sham sessions (*p =* 0.049, S4A Table, Fig 4C), although the amount of increase was greater in cooling sessions (from 150.7 ± 5.6 ms to 162.0 ± 6.2 ms) than in sham sessions (from 151.2 ± 5.6 ms to 158.4 ± 6.2 ms). Due to a smaller sample size (*n* = 30 sessions) compared with the previous analysis using 15-trial blocks (*n* = 192 blocks) and a reduced statistical power (0.18 versus 0.52 in previous analysis), this subtle effect of cooling on prosaccade SRTs did not achieve statistical significance like it did in the previous analysis (Fig 2B, lower panels). In antisaccade trials, we found a significant main effect of session type (*F*_1,56_ = 4.27, *p =* 0.043) and epoch × session type interaction (*F*_1,56_ = 8.60, *p =* 0.0049; S4B Table, Fig 4D). While the SRTs were equivalent in baseline epochs across session types (post hoc Tukey’s test, *p =* 1.0), they were significantly longer in cooling epochs than in control epochs (*p =* 0.008). SRTs also increased with epoch (*F*_1,56_ = 51.6, *p =* 1.7 × 10^−9^) in both session types (cooling: *p =* 0.00016; sham: *p =* 0.018). Unlike in response accuracy, we did not find any effect of post-switch stage (main effect: *F*_1,56_ = 0.48, *p =* 0.49; 3-way interaction: *F*_1,56_ = 0.05, *p =* 0.82). Thus, similarly across post-switch stages, ACC deactivation increased the animals’ SRTs under the antisaccade rule beyond the effect of epoch per se.

Taken together, dACC deactivation impaired performance in the Stable but not Early stage after prosaccade-to-antisaccade switches; it also increased the SRTs in post-switch antisaccades across both stages. It is possible that a floor effect accounted for the lack of effect of cooling during the Early stage, given that the performance improved significantly at the Early to Stable transition for each rule, epoch, and session type (repeated-measures ANOVA described above and shown in S3 Table, post hoc Tukey’s test: *p ≤* 0.00015 in all cases). While appearing normal in their ability to switch to the new rule, the animals showed deficits in maintaining it or using it to achieve the same level of performance as before dACC deactivation.

### dACC deactivation altered oscillatory activities in the dlPFC

We started our analysis of the LFP data with an analysis of the effect of epoch and dACC deactivation on dlPFC oscillations during intertrial intervals (ITIs; −1.15 to −0.05 s from fixation onset) and fixation periods (0 to 1.1 s from fixation onset) separately. To do this, we decibel normalized (see Materials and methods) the LFP power from cooling or control epochs against that from baseline epochs, respectively, for ITIs and fixation periods. In sham sessions, this quantified the epoch-related change in LFP power; in cooling sessions, this difference was induced by both epoch and dACC deactivation. We then performed a set of 16 (4 frequency bands × 2 session types × 2 task periods) one-sample *t* tests to compare these power differences to zero, with adjustments for family-wise false discovery rate [49,50]. For ITIs, we found that dACC deactivation resulted in a significant increase in theta (4–8 Hz) activity and decrease in low (13–20 Hz) and high beta power (21–30 Hz; one-sample *t* tests with adjusted *p*-values: theta: *t*_95_ = 5.95, *p =* 2.4 × 10^−7^; low beta: *t*_95_ = −8.42, *p =* 3.1 × 10^−12^; high beta: *t*_95_ = −5.26, *p =* 2.8 × 10^−6^), contrasted with a lack of change in sham sessions in each frequency band (corrected *p* ≥ 0.072). Similarly, in fixation periods, dACC deactivation increased theta power and decreased LFP power in both beta bands; it also reduced alpha power (theta: *t*_95_ = 5.95, *p =* 2.4 × 10^−7^; alpha: *t*_95_ = −8.42, *p =* 3.1 × 10^−12^; low beta: *t*_95_ = −8.42, *p =* 3.1 × 10^−12^; high beta: *t*_95_ = −5.26, *p =* 2.8 × 10^−6^). In sham sessions, epoch itself resulted in a reduction in low beta power and an increase in high beta power (low beta: *t*_95_ = −2.25, *p =* 0.047; high beta: *t*_95_ = 2.42, *p =* 0.034), although these changes were relatively small (mean ± SEM: −0.12 ± 0.053 and 0.092 ± 0.038) compared with those found in cooling sessions (−0.91 ± 0.096 and −0.68 ± 0.13). In short, dACC deactivation affected the LFP power during both task and nontask periods in a frequency-dependent fashion.

We also conducted a repeated-measures ANOVA of epoch-related changes in LFP power, with task (fixation versus ITI), session type, and frequency bands as within-subject variables, given that the LFPs were obtained from the same channels at different time or different frequencies. We found a main effect of both session type (greater reduction in cooling sessions, mean ± SEM: −0.28 ± 0.11 versus −0.0069 ± 0.059, *F*_1,95_ = 5.08, *p =* 0.026) and task (greater reduction in task: −0.20 ± 0.057 versus −0.094 ± 0.065, *F*_1,95_ = 113.68, *p <* 4.9 × 10^−324^), as well as an interaction between the two, indicating a difference in the effect of cooling during task and nontask periods (*F*_1,95_ = 8.09, *p =* 0.0055; S5 Table). Thus, normalizing fixation-period activity against the ITI power would be necessary to reveal any effect of dACC deactivation that went beyond its effect on the ITIs and was specific to the task.

### dACC deactivation altered task-related oscillations in the dlPFC

Because the interaction between dACC and dlPFC may be critical to the allocation and regulation of cognitive control demanded by the task [14], dACC deactivation was expected to alter task-related oscillatory activities in the dlPFC. To isolate the task-related aspect of local oscillations, for each channel we normalized the spectral power of fixation-period LFP against the LFP power during non-task periods—the ITIs. Such within-channel normalization is also necessary before activities can be combined across channels and animals.

**Fig 5:**
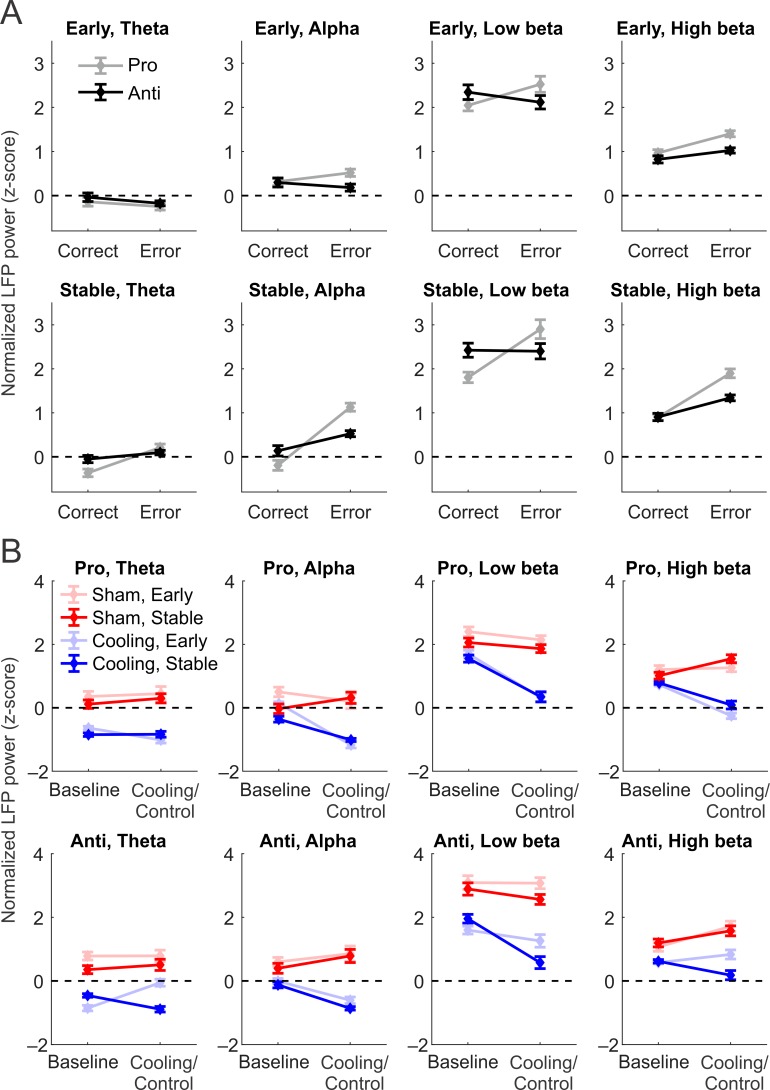
Task-related LFP power in theta (4–8 Hz), alpha (9–12 Hz), low beta (13–20 Hz), and high beta (21–30 Hz) frequency bands in correct trials under different rules and post-switch stages. In each frequency band, LFP power during fixation periods was standardized against LFP power during ITIs. See S6 and S7 Tables for complete sets of result from statistical tests. (A) Averaged normalized LFP power preceding correct (left symbols in each plot) versus error (right symbols) responses at the Early (upper panels) versus Stable (lower panels) post-switch stage, under the prosaccade (gray) and antisaccade (black) rule. Only data from the baseline epochs were included. Significantly positive z-scores were observed in all but theta band (first panels in both rows), suggesting task-related activities in alpha and beta bands (all other panels). In low beta band (third upper and lower panels) at both stages and theta and alpha bands at the Stable stage (first and second lower panels), there is an interaction between rule and performance: in correct trials, LFP power was stronger under the antisaccade rule, but in erroneous antisaccade trials, the LFP power was weaker, which demonstrates rule representation. (B) Averaged normalized LFP power during correct trials only, during baseline (left symbols in each panel) and cooling/control epochs (right symbols) in sham (red) and cooling (blue) sessions, at Early (light red/blue) and Stable (dark red/blue) stages and under prosaccade (upper panels) and antisaccade (lower panels) rules. Interaction between epoch (left versus right) and cooling (blue versus red) during at least one post-switch stage was found in all but theta band under the prosaccade rule (first upper panel), which reflected an effect specific to cooling rather than an epoch effect that may be found in both session types. This effect was exacerbated in antisaccade trials in theta and both beta bands (first, third, and fourth lower panels) at the Stable stage (dark blue) compared with the Early stage (light blue), indicating a stage-specific reduction in task-related activities. Data associated with this figure can be found at 10.6084/m9.figshare.8236589. LFP, local field potential; ITI, intertrial interval.

Before investigating the effect of epoch and cooling, we first examined whether the power of task-related LFPs differed by task rule and performance using baseline periods of both sham and cooling sessions combined. For each channel and in each frequency band (theta: 4–8 Hz, alpha: 9–12 Hz, low beta: 13–20 Hz, high beta: 21–30 Hz), the LFP power during the fixation period was z-score normalized against the mean and standard deviation of the LFP power from ITIs combined across all conditions (Fig 5A). We performed a 4-way repeated-measures ANOVA on baseline LFP power, with frequency band (theta, alpha, low beta, and high beta), post-switch stage (Early versus Stable), rule (prosaccade versus antisaccade), and performance (correct versus error) as within-subject variables. We found main effects of all 4 factors (S6 Table). Among the frequency bands, low beta was the strongest task-related rhythm, followed by high beta and alpha (*F*_1,95_ = 108.93, *p <* 4.9 × 10^−324^). Theta-band power was not significantly different from zero, indicating a lack of task-related theta activity in the baseline epochs (one-sample *t* test with family-wise error correction: theta: *t*_95_ = −1.20, *p =* 0.23, other bands: *t*_95_ ≥ 4.39, *p ≤* 3.9 × 10^−5^). Overall, the Stable post-switch stage had stronger LFP power than the Early stage (*F*_1,95_ = 49.78, *p =* 2.8 × 10^−10^), and correct trials had stronger LFP power than errors (*F*_1,95_ = 37.46, *p =* 2.1 × 10^−8^). Several subplots in Fig 5A demonstrated an interactive effect between rule and performance (*F*_1,95_ = 159.17, *p <* 4.9 × 10^−324^): in correct trials (left symbols in each panel), antisaccades were preceded by stronger fixation-period LFP power (black) than prosaccades (gray), whereas in error trials (right symbols), this pattern was reversed. This interaction indicated that LFP power contained rule representations, which predicted error responses when mismatched with the current rule. This phenomenon was observed in low beta band at both Early and Stable post-switch stages (post hoc Tukey’s test, *p =* 2.8 × 10^−5^ in all cases), as well as in both theta and alpha bands during the Stable stage only (*p*-values between 2.8 × 10^−5^ and 2.9 × 10^−5^). At the Early stage, antisaccades were preceded by stronger theta power than prosaccades whether in correct or error trials (*p =* 5.0 × 10^−5^ and 0.038, respectively) and preceded by alpha power equivalent to those in prosaccade trials (*p =* 1.0), suggesting a lack of rule representation in these frequency bands at the Early stage. High beta power was stronger under the prosaccade than the antisaccade rule during both correct and error trials at the Early stage (*p =* 2.8 × 10^−5^ for both) and was not different in correct trials under the 2 rules at the Stable stage (*p =* 1.0), suggesting a lack of rule representation. In short, while low beta-band oscillatory power contains rule representation at both Early and Stable stages, theta- and alpha-band power represented the rule only during the Stable post-switch stage. The finding that alpha and/or low beta power was stronger in antisaccade than in prosaccade trials was consistent with previous observations in the frontal eye field using a similar antisaccade task in the common marmoset [51].

After establishing a role of low-frequency oscillations in rule representation, we investigated the effect of cooling and epoch on task-related LFP power during correct trials (Fig 5B). We conducted a 5-way repeated-measures ANOVA, with frequency band, rule, post-switch stage, cooling, and epoch all as within-subject variables. The analysis revealed significant main effects for all five factors (S7 Table). Additionally, we found a significant epoch by session type interaction (*F*_1,95_ = 33.5, *p =* 9.3 × 10^−8^): while task-related LFP power remained the same from baseline to control epochs in sham sessions (post hoc Tukey’s test, *p =* 0.50), it decreased significantly from baseline to cooling epochs in cooling sessions (*p =* 0.00014). This demonstrated an effect of cooling in the absence of any effect of epoch. Notably, LFP power decreased from Early to Stable stage (*F*_1,95_ = 133.20, *p <* 4.9 × 10^−324^), an effect observed in each frequency band under each rule (*p =* 2.9 × 10^−5^ in all cases). The effect of stage also interacted with the effect of epoch and session type (3-way interaction: *F*_1,95_ = 36.4, *p =* 3.1 × 10^−8^): in sham sessions, while Early-stage LFP power remained unchanged from baseline to control epochs (post hoc Tukey’s test, *p =* 0.31), Stable-stage LFP power increased significantly (*p =* 0.00012). This increase may reflect an enhancement in task-related processes with the accumulation of experience or a compensatory process against fatigue or satiation over the reward. In cooling sessions, however, both Early-stage and Stable-stage LFP power decreased from baseline to cooling epochs (*p =* 0.00012 in both stages). Thus, at the Early stage, the negative impact of dACC deactivation on dlPFC LFP power was observed with no effect of epoch per se, whereas at the Stable stage, this negative impact likely canceled out the enhancing effect of epoch and further reduced the task-related oscillatory activities.

We then examined the 2 rules separately (rule × stage × session type × epoch 4-way interaction: *F*_1,95_ = 42.6, *p =* 3.3 × 10^−9^). At the Stable stage, LFP power increased with epoch (post hoc Tukey’s test, *p =* 0.00015 and 0.038 for pro and anti) and decreased with cooling (*p =* 0.00015 for both) under both rules. By contrast, at the Early stage, LFP power did not change with epoch (*p =* 0.49) and decreased with cooling in prosaccade trials (*p =* 0.00015), whereas in antisaccades, it increased with epoch (*p =* 0.00015) and remained the same with cooling (*p =* 0.36). Thus, the contrast between cooling and sham sessions was stronger at the Stable than the Early post-switch stage.

Each frequency band demonstrated these effects to a different extent (5-way interaction: *F*_3,285_ = 58.2, *p <* 4.9 × 10^−324^, Fig 5B). In Stable-stage antisaccade trials, the contrast between cooling-induced decrease and epoch-related increase was observed in theta, alpha, and high beta bands (post hoc Tukey’s test, *p* between 0.0021 and 2.7 × 10^−5^). In Stable-stage prosaccade trials, this epoch × session type interaction was found in alpha and high beta bands (Fig 5B, second and fourth panels in both rows, dark-colored symbols). Theta power increased with epoch in sham sessions but remained unchanged in cooling sessions (Fig 5B, upper row, first panel, dark-colored symbols). Because low beta power decreased with epoch in both session types and under both rules (*p =* 2.7 × 10^−5^ for all cases, Fig 5B, third panel in both rows, dark-colored symbols), the effect of cooling in low beta remained unclear.

During the Early post-switch stage, the changes with epoch or cooling were much more heterogeneous. In prosaccade trials, while cooling reduced theta and high beta power (*p =* 2.7 × 10^−5^ in both cases) compared with a lack of effect of epoch alone in sham sessions (*p =* 0.82 and 1.0; Fig 5B, upper row, first and fourth panels, light red versus light blue symbols), alpha and low beta power decreased with epoch in both session types (*p =* 2.7 × 10^−5^ in all cases; Fig 5B, upper row, second and third panels, light-colored symbols). In antisaccade trials, alpha-band power increased with epoch and decreased with cooling (*p =* 2.7 × 10^−5^ in both cases)—a pattern of change that resembled those observed in the Stable stage (Fig 5B, lower row, second panel, light-colored symbols). Meanwhile, theta-band power did not change with epoch but increased with cooling (*p =* 1.0 and 2.7 × 10^−5^, respectively; Fig 5B, lower row, first panel, light-colored symbols), and low beta power did not change with epoch but decreased with cooling (*p =* 1.0 and 2.7 × 10^−5^), whereas high beta power increased with both epoch and cooling (*p =* 0.0018 and 2.7 × 10^−5^, respectively). The heterogeneous patterns of change in LFP power may reflect multiple beneficial or deleterious processes at work, potentially contributing to the overall lack of change with either epoch or cooling in performance at the Early stage (Fig 4).

Overall, we have found a cooling-related decrease in task-related LFP power, contrasted with an increase or lack of change with epoch only, in 2 or more frequency bands for both Early and Stable stages and under both rules. This confirmed a deleterious effect of dACC deactivation on task-related oscillatory activities in the dlPFC. The loss of task-related oscillatory power in correct trials may contribute to the increase in SRTs, which was observed across stages and rules (Figs 2, 4C and 4D). Additionally, in both theta and high beta band, the effect of dACC deactivation was specific to antisaccade trials and to the Stable post-switch stage. This effect coincided with the specific impairment in performance during the Stable stage under the antisaccade rule and may reflect a functional role of dlPFC theta and high beta activities in rule maintenance and their dependence on dACC activity. One limitation of this analysis was the difficulty in determining the effect of dACC deactivation when task-related activities were weakened in both session types, e.g., in the low beta band (Fig 5B, third panels in both rows).

**Fig 6:**
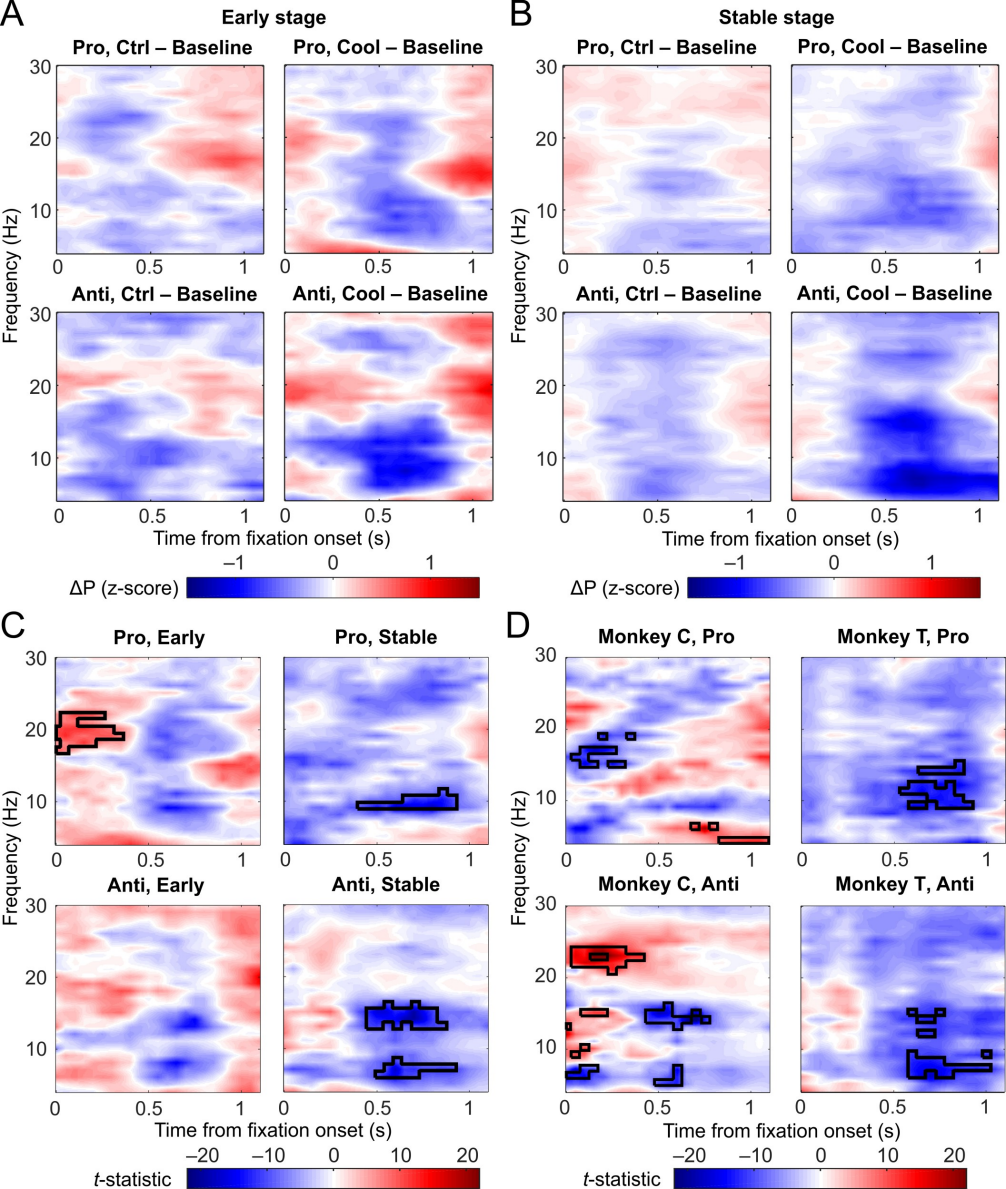
Difference in normalized, time-resolved LFP power between cooling/control epochs and baseline epochs. In (A) and (B), blue color indicates a decrease in LFP power from baseline to cooling/control epochs, and red color indicates an increase in LFP power. (A) Effects of epoch alone in sham sessions (left panels) and of epoch and cooling in cooling sessions (right panels) at the Early stage. Cooling sessions seemed to have a greater decrease in theta and alpha/low beta band compared with sham sessions, as indicated by the darker shade of blue. (B) Effects of epoch alone in sham sessions (left panels) and of epoch and cooling in cooling sessions (right panels) at the Stable stage. Cooling sessions appeared to have a greater decrease in theta and alpha/low beta band compared with sham sessions, as indicated by the darker shade of blue, especially among antisaccade trials (bottom right panel). (C) Maps of *t*-statistics for corresponding comparisons in (A) and (B). Top left panel shows *t*-statistics between sham and cooling sessions under the prosaccade rule at the Early stage (upper panels in [A]), and bottom left panel contains *t*-statistics between session types under the antisaccade rule at the Early stage (lower panels in [A]). Top right panel shows *t*-statistics between sham and cooling sessions under the prosaccade rule at the Stable stage (upper panels in [B]), bottom right panel contains *t*-statistics between session types under the antisaccade rule at the Stable stage (lower panels in [B]). Black contours denote significant differences according to the permutation test. The red area with black contour in the top left panel indicates an increase LFP power in cooling sessions coinciding with a decrease in sham sessions. The blue areas in the Stable-stage power spectra (right panels) indicate a significantly greater decrease in cooling sessions than in sham sessions. (D) Maps of *t*-statistics between cooling and sham sessions at the Stable stage for each subject. In prosaccade trials (upper panels), only Monkey T had significant cooling-related decrease (black contour indicates *p <* 0.001 by permutation test) in alpha/low beta band during mid-to-late fixation period. In antisaccade trials (lower panels), both monkeys had significant cooling-related decrease in theta and low beta bands during the same period of fixation. Data associated with this figure can be found at 10.6084/m9.figshare.8236589. Ctrl, control; LFP, local field potential.

To better isolate the effect of dACC deactivation, and to examine its timing and duration, we constructed time-resolved power spectra for task-related activities (fixation period power normalized against ITIs), then subtracted the power spectra of LFPs in the baseline epochs from those in the cooling or control epochs for each type of session (Fig 6A and 6B). In Fig 6A and 6B, red color indicates an increase from the baseline to the cooling/control epochs, whereas blue color indicates a decrease. While some power reduction with epoch is visible in sham sessions (Fig 6A and 6B, left columns), this change was stronger in cooling sessions as indicated by the darker shade of blue in theta, alpha, and low beta bands in the right columns of Fig 6A and 6B. Now that the plots reflect the effect of epoch, we needed only to test for the effect of session type. This was carried out with a nonparametric cluster-based permutation test (based on paired *t*-statistic; see Materials and methods). A map of the *t-*statistics between the epoch-related difference in sham sessions (Fig 6A, top left panel) and the cooling- and epoch-related difference in cooling sessions (Fig 6A, top right panel) during prosaccade trials at the Early stage is shown in the top left panel of Fig 6C. In parallel, the bottom left panel of Fig 6C shows the *t-*statistic map for the between-session comparison during antisaccade trials at the Early stage (compare bottom panels, Fig 6A). The top right panel of Fig 6C shows the *t-*statistics for the comparison in prosaccade trials at the Stable stage (compare top panels, Fig 6B), and the bottom right panel shows *t-*statistics for the comparison in antisaccade trials at the same stage (compare bottom panels, Fig 6B). All 4 comparisons were conducted in one cluster-based permutation test to avoid the issue of multiple comparisons, and only the strongest effects would survive the test. At the Stable stage, a significant effect of cooling was found in alpha power (8–12 Hz) in prosaccade trials (Fig 6C, top right panel) and in theta and alpha power (between 6 and 16 Hz) in antisaccade trials (bottom right panel; black contour indicates significant combinations in frequency and time, *p* < 0.001). The effects of cooling on the Early-stage LFP power (shown in Fig 5B) was not as strong as those at the Stable stage and did not reach significance (Fig 6C, left column). We then verified the Stable-stage effect in each monkey (Fig 6D). For Monkey C, cooling reduced low-beta power (15–19 Hz) early in fixation in prosaccade trials and reduced theta and alpha power (between 5 and 16 Hz) in the middle of the period (Fig 6D, left column, black contour indicates *p* < 0.001). For Monkey T, cooling reduced alpha power (9–16 Hz) in prosaccade trials and reduced theta and alpha power (6–16 Hz) in antisaccade trials, in both cases during the second half of the fixation period (Fig 6D, right column, black contour indicates *p* < 0.001). Hence, a cooling-induced reduction in LFP power was observed in both monkeys and was more consistent in frequency and timing in antisaccade than prosaccade trials across subjects. Notably, a decrease in task-related theta activity (5–9 Hz) was found specifically in Stable-stage antisaccade trials in both monkeys (Fig 6D), consistent with results from the a priori analysis (Fig 5B) and coinciding with the impairment in performance.

In summary, while dACC deactivation had a negative impact on task-related oscillatory activities across rules, post-switch stages, and frequency bands, this effect was especially strong at the Stable stage, starting halfway through the fixation period, and was in a range between 6 and 16 Hz. It was also more consistent across subjects under the antisaccade rule. This effect of dACC deactivation on task-related oscillations was found despite its general enhancing effect on theta-band power in task and nontask periods. This effect also weakened or strengthened as the animal entered the Early or the Stable stage after each rule switch.

Compared with the previous analysis, which focused on averaged power across the fixation period (Fig 5B), time-resolved power spectra were more sensitive to strong effects with limited temporal duration. This may explain why the negative impact of dACC deactivation on beta activities between 17 and 30 Hz was not detected in this analysis, although the effect is still clearly visible (Fig 6C and 6D, right panels). Considering the results from both a priori and time-resolved spectral analyses, it was clear that, while task-related alpha activity was affected during both rules and both stages, task-related theta activity was reduced by dACC deactivation specifically in the Stable-stage antisaccade trials.

### dACC deactivation reduced C-E difference in beta activities at the Stable stage

In the previous section, we reported task-related alpha and low beta activities that were stronger preceding correct antisaccades and incorrect antisaccades under the prosaccade rule (Fig 5A). Now that we have shown that dACC deactivation altered dlPFC LFP power in a rule- and stage-dependent manner (Figs 5B and 6), we wondered whether it additionally affected dlPFC activities in correct and error trials differently, i.e., whether the effect of dACC deactivation depended on task performance.

First, we conducted a 6-way repeated-measures ANOVA on LFP power, using frequency band, stage, rule, performance (correct versus error), session type, and epoch as within-subject variables, given that the LFPs were obtained from the same set of channels. We found significant main effects of performance (correct < error, *F*_1,95_ = 33.2, *p =* 1.0 × 10^−7^), epoch (baseline > control/cooling, *F*_1,95_ = 90.2, *p =* 2.0 × 10^−15^), and session type (sham > cooling, *F*_1,95_ = 74.3, *p =* 1.5 × 10^−13^), but the 3 factors did not interact (*F*_1,95_ = 0.39, *p =* 0.54; S8 Table). Post hoc Tukey’s test revealed a similar epoch-related decrease in LFP power in both correct and error trials (post hoc Tukey’s test: *p =* 0.00012 for both) in cooling sessions, contrasted with an epoch-induced increase in correct trials in sham session (*p =* 0.0019). LFP power in error trials did not change in sham sessions (*p =* 1.0). These results suggested that dACC deactivation did not have very different effects on the LFP power during correct and error trials. This finding is also evident upon a comparison between Fig 5B, which shows results from correct trials, and S5 Fig, which shows results from error trials.

Across the 2 task rules, 2 post-switch stages, and 4 frequency bands, which gave rise to a total of 16 scenarios, dACC deactivation appeared to affect correct and error trials similarly across most of these scenarios, despite a 6-way interaction involving all factors (*F*_1,95_ = 102.9, *p <* 4.9 × 10^−324^). Specifically, in 13 out of the 16 scenarios (2 exceptions with theta and 1 with high beta power in Early-stage antisaccade trials), we found a decrease in LFP power in correct trials in cooling sessions (all cases: *p <* = 6.2 × 10^−5^). In 11 out of these 13 scenarios, we also found a significant decrease in LFP power during error trials (all cases: *p ≤* 6.2 × 10^−5^). The only 2 exceptions were in the theta band, in prosaccade trials at the Early stage, and in antisaccade trials at the Stable stage, where LFP power during error trials increased with cooling contrasted with a decrease in correct trials (all cases: *p =* 6.2 × 10^−5^). Thus, dACC deactivation reduced task-related alpha and beta powers in a similar fashion in both correct and error trials.

Despite the similar effect of dACC deactivation across correct and error trials, it remained unclear whether this change rendered the 2 trial types more similar or more different in task-related LFP power. If the difference in fixation-period LFP power between correct and error trials (Fig 5A) at baseline was reduced by cooling, then this could have contributed to the impairment in performance. It should be noted that a reduction in task-related activities during both correct and error trials did not necessitate a smaller difference between the two, because the normalized LFP power could be a negative number (if activity level was lower during the task) and was free from any floor effect.

**Fig 7:**
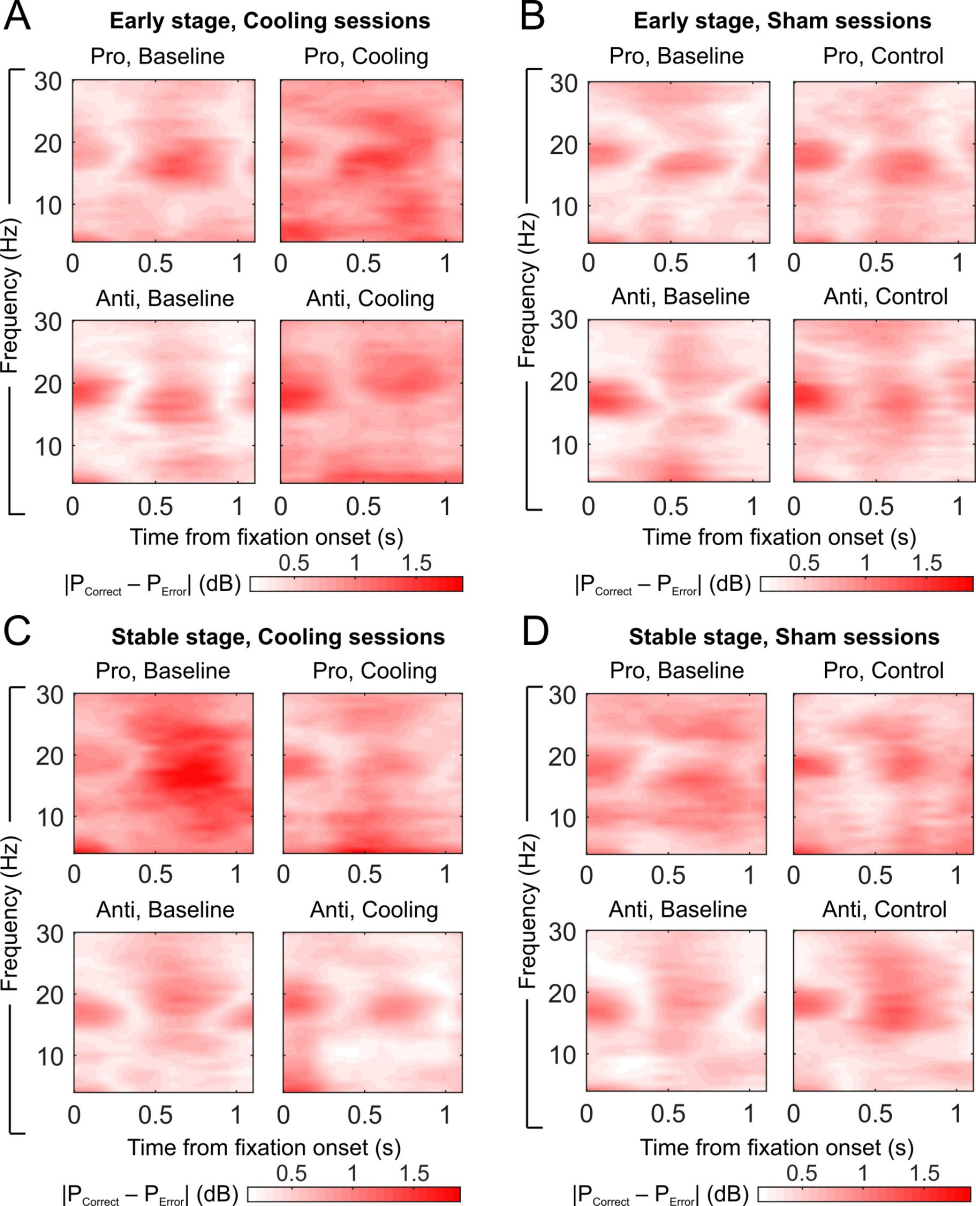
Effects of epoch and dACC deactivation on absolute C-E difference in LFP power. Within each subplot, upper panels show absolute C-E difference under the prosaccade rule, and lower panels show the difference under the antisaccade rule. Left panels show the difference in the baseline epoch, while right panels are based on the cooling/control epoch. The subplots show absolute C-E difference in (A) Early stage in cooling sessions, (B) Early stage in sham sessions, (C) Stable stage in cooling sessions, and D) Stable stage in sham sessions. Data associated with this figure can be found at 10.6084/m9.figshare.8236589. C-E, correct-error; dACC, dorsal anterior cingulate cortex; LFP, local field potential.

**Fig 8:**
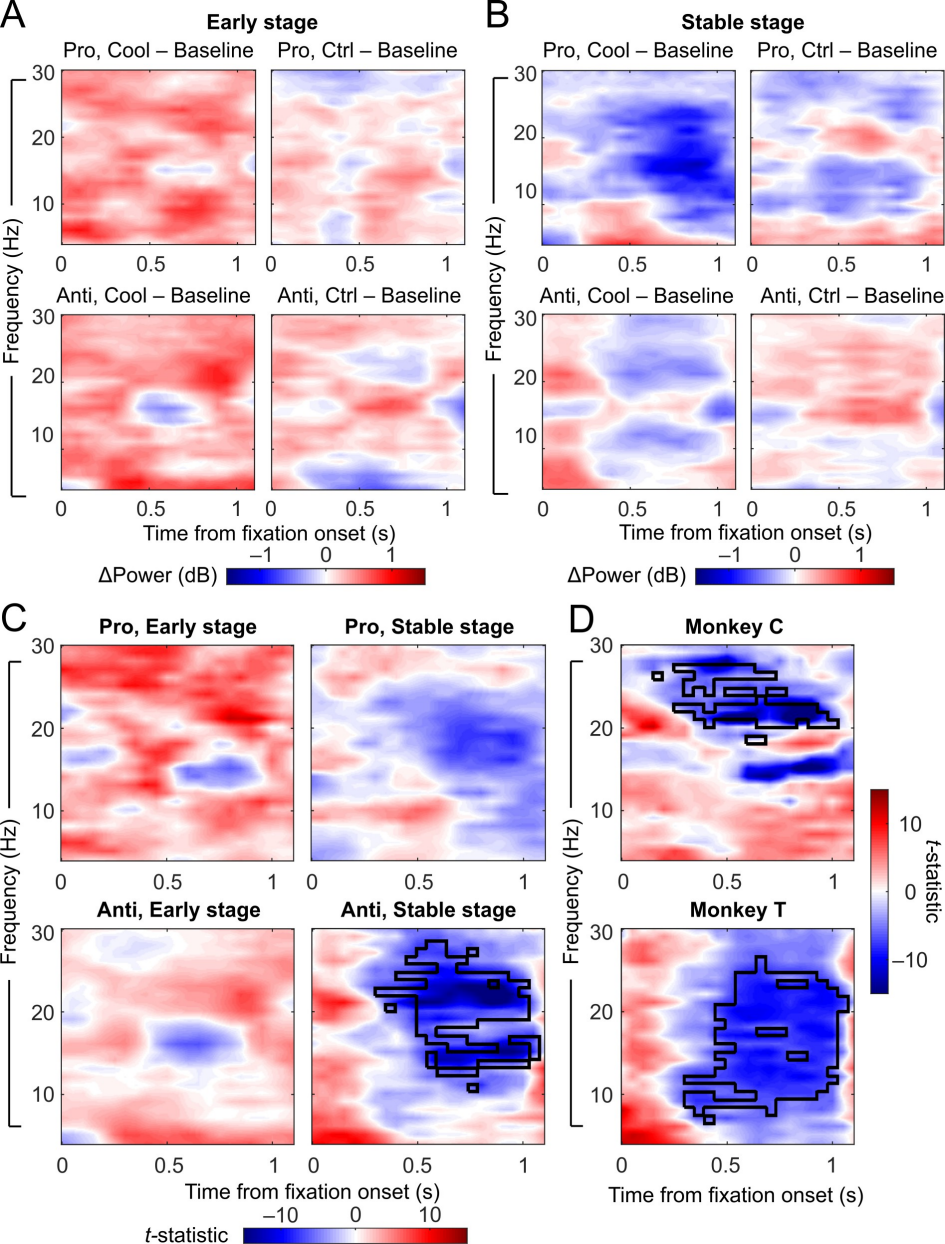
ACC deactivation reduced the C-E difference in dlPFC beta-band LFP power in the fixation periods of antisaccade trials at the Stable stage. (A) Change in C-E difference in LFP powers from the baseline to the cooling epochs (left panels) or the control epochs (right panels) at the Early stage. Left panels demonstrate the effect of both cooling and epoch, while right panels illustrate the effect of epoch only. (B) Change in C-E difference from the baseline to the cooling epochs (left panels) or the control epochs (right panels) at the Stable stage. (C) *t*-statistics between the changes due to cooling and epoch combined and those due to epoch alone, which demonstrate the effect of cooling. Left panels: *t*-statistics on the effect of cooling in prosaccade (upper panels) and antisaccade (lower panels) trials at the Early post-switch stage. Right panels: *t*-statistics on the effect of cooling at the Stable stage. Cooling specifically reduced the C-E difference in beta-band activities in antisaccade trials at the Stable stage (black contour indicates *p <* 0.001 by permutation test, bottom right panel) by a cluster-based permutation test considering both rules and stages. (D) The effect of cooling on the C-E difference in beta-band activities in antisaccade trials at the Stable stage was found in each of the 2 animals. Black contours mark the area of significance by a cluster-based permutation test. Data associated with this figure can be found at 10.6084/m9.figshare.8236589. ACC, anterior cingulate cortex; C-E, correct-error; Ctrl, control; dlPFC, dorsolateral prefrontal cortex; LFP, local field potential.

We computed the absolute difference in LFP power spectra between correct and error trials (Fig 7) and then examined how the C-E distance changed with epoch and cooling (Fig 8). For this analysis, we used the time-resolved power spectra to better understand the timing and duration of the effect of dACC deactivation within the fixation period and to capture effects that may not line up well with a priori frequency bands. The effect of dACC cooling appeared to differ across epochs (Fig 7, within 7A–7D, left versus right panels), across session type (Fig 7A versus 7B and 7C versus 7D), across rules (all panels, upper versus lower row), and across post-switch stages (Fig 7A versus 7C and 7B versus 7D). At the Early stage, the difference in LFP power between correct and error trials went through a visible increase from the baseline to the cooling epochs under both rules (Fig 7A, left versus right panels). In sham sessions, the C-E distance also appeared to increase but to a less extent compared with cooling sessions (Fig 7B). At the Stable stage, in the cooling sessions, the C-E distance showed a visible decrease from baseline to cooling epochs under both rules (Fig 7C), whereas in the sham sessions, no such effect of epoch was seen (Fig 7D). If anything, the C-E distance under the antisaccade rule appeared to increase with epoch in the sham sessions (Fig 7D, lower panels).

We then quantified these epoch-related change in C-E distance in cooling and sham sessions, respectively, by subtracting the C-E distance in LFP power during baseline epochs from cooling/control epochs. Specifically, the power spectra shown in the upper right panel minus the one in the upper left panel in Fig 7A gave rise to the upper left panel of Fig 8A, which represents a combined effect of both cooling and epoch on C-E distance in prosaccade trials at the Early stage. In parallel, the difference between the lower panels in Fig 7A is shown in the lower left panel of Fig 8A, which represents the combined effect in antisaccade trials. For the sham sessions, the difference between the upper panels of Fig 7B is shown in the upper right panel of Fig 8A, and the difference between the lower panels of Fig 7B is shown in the lower right panel of Fig 8A. Because cooling led to a visible increase in C-E distance (Fig 7A, left versus right panels), the epoch-related change in cooling sessions was positive, as indicated by the red color (Fig 8A, left panels); the same is not seen for the sham sessions (right panels). The same procedure was repeated for the Stable stage: Fig 8B was calculated from power spectra shown in Fig 7C and 7D. The decrease in C-E distance from baseline to cooling epochs under the prosaccade rule at the Stable stage (Fig 7C, upper panels) is now visible as a large blue area (Fig 8B, upper left panel), although some epoch-related decrease in C-E distance is also present in the sham sessions (Fig 8B, upper right panel). In antisaccade trials, although there is no dramatic change, as indicated by the absence of deep red or blue, C-E distance changed in the opposite directions in cooling versus sham sessions: while in sham sessions it increased somewhat with epoch as shown by the red hue, dACC deactivation canceled out this positive effect of epoch and brought the change to the negative direction.

To isolate the effect of dACC deactivation, we performed a cluster-based permutation test (using paired *t*-statistics) between the epoch-related changes in C-E distance from the 2 types of sessions. To avoid the statistical pitfall of multiple comparisons, a single permutation test was performed simultaneously for all 4 rule-by-stage groups, in which a dominant effect can overshadow other smaller effects. The results were then overlaid on time-frequency maps of *t-*statistics (Fig 8C) shown as black contours (lower right panel). In Fig 8C, the upper left panel shows the *t-*statistics between data from cooling and sham sessions under the prosaccade rule at the Early stage (Fig 7A, upper panels); the lower left panel shows the results from antisaccade trials at the same stage. The widespread red areas indicate a more positive impact of dACC deactivation on C-E distance compared with epoch per se at the Early stage. The right panels in Fig 8C visualize the session-type effect from the Stable stage under the 2 rules. In the upper right panel, the blue area in the beta frequency range suggests that dACC cooling resulted in a somewhat greater reduction in C-E distance compared with the effect of epoch alone under the prosaccade rule, although this effect did not reach significance in the permutation test. In the lower right panel, significant effect of session type based on the permutation test (black contours, *p* < 0.001) coincided with a dark blue area, which demonstrates a strong negative impact of dACC cooling on C-E distance in both low and high beta bands (13–29 Hz) under the antisaccade rule at the Stable stage. Additionally, this effect did not start until 300 ms after fixation onset and lasted towards the end of the fixation period. This reduction in C-E distance due to dACC deactivation was observed in each of the 2 animals with similar timing and duration (Fig 8D), although in Monkey T it spanned alpha, low beta, and part of the high beta band (lower panel), whereas in Monkey C it was mostly limited to the high beta band (upper panel).

The final comparison between session types was carried out to demonstrate to its full strength an effect that was already visible as an epoch–session-type interaction in the lower panels of Fig 8B. In fact, this effect can be seen directly in the plots of C-E difference across epochs and sessions: while it became stronger from baseline to control epochs in sham sessions (Fig 7D, lower panels), it appeared to grow weaker from baseline to cooling epochs in cooling sessions (Fig 7C, lower panels). To produce this effect in the opposite direction, the negative impact of dACC deactivation must have counteracted the enhancing tendency of epoch per se.

In summary, given that dlPFC LFP power reflected the animals’ rule representation, which differed from the actual task rule in error trials (Fig 6A), we examined the effect of dACC cooling on the oscillatory activities during correct and error trials as well as on the C-E difference in LFP power. Although dACC deactivation affected the task-related LFP power similarly across correct and error trials (Fig 6B versus S5 Fig), it had stage- and rule-dependent effects on the absolute difference between correct and error trials (Fig 8). This performance-related difference in normalized LFP power was reduced in the beta band during antisaccade trials specifically at the Stable but not the Early post-switch stage. While at the Early stage the new rule may yet to gain control over response selection, by the Stable stage, its control was established (Figs 2 and 3) and correlated with a consistent rule representation in the dlPFC (Fig 5A). While this control may lapse and lead to errors, or perhaps the animal decided to test the alternative rule, these responses were expected to be preceded by neural correlates distinct from those preceding correct responses. Weakening of this difference in task-related activity as a result of dACC deactivation may contribute to the impaired antisaccade performance at the Stable post-switch stage.

## Discussion

We combined cryogenic reversible deactivation of the dACC with microelectrode array recordings from the dlPFC to investigate the functional roles of both regions—which are part of an extensive cognitive control network—in an uncued rule-switching task in macaque monkeys. dACC deactivation altered the oscillatory power in the dlPFC in a frequency-dependent manner and weakened task-related activities in theta, alpha, and beta frequency bands. Additionally, it reduced the difference between the oscillatory power preceding correct and error responses under the cognitively demanding antisaccade rule, which coincided with an impairment in behavioral performance at the same stage after each prosaccade-antisaccade rule switch. We will discuss the implication of these findings in the cognitive roles of dACC and dlPFC, respectively, but in an intact brain, the 2 regions likely engage in frequent communication, which may explain why they tend to be coactivated in cognitively demanding tasks [1,2,4,5,52]. Hence, we will end our discussion by speculating how their interaction contributes to cognitive flexibility.

Before discussing the effects of dACC deactivation, it is critical to first address the validity of the cryoloop technique. While we did not implant electrodes to monitor dACC neural activity, previous studies have used measures of 2-deoxyglucose reuptake [53,54] and microelectrode recording [55] to verify the extent of neural deactivation surrounding cryoloops. Given that cortical temperature rises rapidly away from the cryoloop (10°C–20°C/mm) [56] and that evoked neural activities remain normal at above 24°C and stop below 20°C [57], we initially set the target to 1°C–3°C to deactivate as large an area of cortical tissue as possible without reaching subzero temperature and causing tissue damage [58]. At this target temperature, if the cryoloops made immediate contact with the tissue, they were expected to stop neural activities within a 2 mm range from the surface of the loops and to affect the tissue temperature within a 3 mm range, as demonstrated in previous studies [59,60]. While Monkey C was able to continue performing the task at this target temperature, Monkey T stopped task performance completely at temperatures below 14°C–15°C. Although for both monkeys we implanted the cryoloops precisely based on the cingulate and principal sulci, the small variation in the amount of cortical tissue contacted by the loops was difficult to control surgically despite our best effort. We speculate that in Monkey T the cryoloops were in closer contact with the cortical tissue than in Monkey C, therefore cooling to 1°C–3°C would affect a larger tissue volume than expected. Using the higher target temperature thus greatly reduced the likelihood that cooling affected a greater volume in Monkey T than in Monkey C or the possibility that cooling affected tissue outside the dACC. The strong behavioral effect of dACC deactivation in Monkey T was consistent with the role of the dACC in motivation and reward-related behavior [23,24].

As it was not possible to monitor the temperature of the same dlPFC tissue in which the Utah array was implanted, we cannot unequivocally prove that the dlPFC was not directly affected by cooling. This is an inherent limitation in a study that combines cryogenic inactivation and electrode-array recordings. Because our cryoloops measured 8–10 × 3 × 2 mm and were inserted into the cingulate sulcus, given the 3 mm maximal spread of temperature change, the volume of thermally affected cortex was expected to be within a volume with dimensions 14–16 mm anterior-posterior, 9 mm medial-lateral, and 8 mm dorsal-ventral. The lateral spread of 4.5 mm was not expected to thermally affect the array in the dlPFC, which was about 10 mm away, and the impact on neural activity (spread of 2 mm) was not expected to reach beyond 3.5 mm from the center of the loop. Additionally, because the cooling affects neural activities by blocking synaptic transmission [61,62], cryogenic deactivation at these temperatures does not affect fibers of passage within the affected volume [63,64]. In summary, we believe that any changes in dlPFC activities were due to loss of communication with the dACC rather than any direct influence of cooling on the dlPFC itself.

In our task, we identified an Early stage after uncued rule switches, a stage of “rule switching,” during which the animals were likely to switch away from the rule they followed on the previous trial (Fig 3). dACC deactivation did not delay or prolong this Early stage and did not increase or decrease the probability of rule switch during these trials, nor did it impair the overall level of performance or increase the SRTs under either task rule (Fig 3). Instead, during the Stable stage—a stage of “rule maintenance”—following the Early stage, dACC deactivation impaired the animals’ performance under the more cognitively demanding antisaccade rule (Figs 2 and 4). Similar performance impairment under challenging rules was observed in human patients suffering from ACC damage [65,66]. This antisaccade deficit may be related to a loss in the animals’ ability or stamina to exert cognitive effort: on response accuracy, dACC deactivation resulted in a deficit that was not produced by time-related factors in the well-trained animals (Figs 2 and 4B); on SRTs that did increase with time in session, dACC deactivation led to an even greater increase, although this was the case in both post-switch stages (Fig 4D). It should be noted that the animals’ performance deficit was not explained by either motor perseveration or an active employment of a fixed-direction strategy—i.e., always look toward the same direction (S3 Fig)—nor was it explained by an increase in impulsivity. Furthermore, dACC-related impairment was not specific to eye movements, since the same increase in errors after initial success in strategy shifting was observed in a dACC lesion study on reversal learning using hand movements [24]. Our findings are consistent with its role in sustaining effective choices based on reward history [23,24] and in controlling cognitive effort [26,28,29], although they do not preclude the involvement of dACC in feedback processing [14,18].

dACC deactivation had profound effects on dlPFC oscillatory activities. While it reduced the power of beta rhythms (13–20 Hz) during both task and nontask periods, this effect was stronger during the task than in ITIs (Fig 5B). Although dACC deactivation enhanced theta-band (4–8 Hz) power in both task and nontask periods, the decrease in task-related activity was even more prominent in theta and alpha (6–16 Hz) than in beta bands (Fig 6). Thus, effects of dACC deactivation on dlPFC oscillatory power could be dissociated from its influence on task-related activities in the dlPFC. What is more, the strength of these effects depended on the post-switch stage: it weakened after a rule switch and strengthened as the animals entered the stage of rule maintenance (Fig 6). Finally, while the reduction in task-related low-frequency oscillations was observed across post-switch stages to different extent (Fig 5B), a significant decrease in the difference in beta-band power (13–29 Hz) between correct and error trials was found only at the Stable stage under the antisaccade rule (Figs 7 and 8). Both animals also displayed a decrease in task-related theta activity (5–9 Hz) uniquely in the Stable-stage antisaccade trials (Fig 6D). Both of these rule- and stage-dependent effects of dACC deactivation coincided with the animals’ impairment in performance maintenance under the antisaccade rule. Because the task rule was encoded in a performance-dependent manner in dlPFC low beta (13–20 Hz) activities immediately after rule switches and persisted into the Stable stage (Fig 5A), we believe that this reduction in C-E difference was relevant to task performance. Thus, while task-related theta and alpha activities were strongly affected during the maintenance of the rules (Fig 6), beta activities appeared to be more strongly tied to behavioral performance. We will discuss the potential functions of beta and theta/alpha rhythms separately.

Correlation between changes in beta activities and performance has been reported in several studies [45,47,67]. In working memory, prefrontal beta oscillations are believed to support the maintenance or (re)activation of the current rule [44,68–70]. In our uncued task, such processes were important both within and across trials in a block between rule switches, which may explain the strong task-related increase in both low and high beta-band power, contrasted with the smaller increase in alpha and lack of change in theta activities (Fig 5A). While the actual content of the task rules are likely encoded in spiking activities, prefrontal beta may support the process by gating information coding and inhibiting potential interference [71,72]. Because antisaccade performance tended to suffer from the interference of the prepotent prosaccade rule, the enhanced low-beta power before correctly performed antisaccades compared with prosaccades may help inhibit such interference during both rule switching and rule maintenance (Fig 5A). During dACC deactivation, both trial types lost task-related beta power, and the difference in power between correct and error trials also decreased, in contrast with an increase in this difference in sham sessions (Fig 8B, lower panels). If prefrontal beta activities were involved in interference inhibition, its weakening in antisaccade trials would lead to an impairment in performance, which was observed here (Figs 2 and 4). Although a decrease in C-E difference was found for the prosaccade trials as well, because the interference inhibition was not as important for this rule, the reduction in task- and performance-related activity did not result in a performance deficit. In short, our findings support a role of prefrontal beta activities in information gating and interference inhibition that are critical in rule maintenance. We speculate that this suggested function of prefrontal beta rhythm is potentially linked to the role of dACC in allocating cognitive effort [25–31]. That is, dACC may be responsible for detecting the need for cognitive effort such as interference inhibition, which then took place in the dlPFC upon receiving communication from the dACC.

dACC deactivation also resulted in a reduction in task-related activities in both theta and alpha band (6–16 Hz) in the dlPFC, which started approximately 400 ms after fixation onset, lasted for several hundred milliseconds, and was especially strong during rule maintenance (Fig 6). For theta band (6–8 Hz, Fig 6C) the reduction was found only during the maintenance of the antisaccade rule—an effect also stood out when the frequency bands were defined a priori (Fig 5B). The dACC is the primary source of “frontal midline theta” activities in electroencephalogram (EEG) studies, which are believed to reflect signals of cognitive control and negative affect [73]. Additionally, dACC theta was found to carry rule information prior to the onset of the stimulus that determines the direction of the saccade [74]. Because task information can be transferred from the dACC to the prefrontal cortex through theta activities [75], the rule-specific reduction in task-related theta activities in the dlPFC may reflect a loss of theta entrainment from the dACC, which conveyed the cognitive control signal [38] more critical for the antisaccade than the prosaccade rule. This finding supports a key role of dACC in task-related prefrontal theta and a role of this interaction in cognitive control. It also suggests that the antisaccade deficit consistently observed in patients with schizophrenia [76–80] may be linked to their attenuated frontal midline theta [42] and that treatments such as direct current stimulation [43], which restore medial-frontal cortical synchrony, may rescue the deficit.

For alpha band, dACC deactivation-induced reduction in task-related decrease was found for both rules (Fig 6). This change in dlPFC neural activity coincided with the increase in reaction times that was also observed across rules and during both post-switch stages (Figs 2 and 4). Similar to the proposed function of beta-band oscillations, alpha-band activities have been suggested to play a role in inhibiting the irrelevant, prepotent rule [51,81,82]. It may be speculated that a loss of task-related alpha activities could lead to greater competition from the alternative rule, thereby reducing the animals’ confidence over their choice and increasing their reaction times. Alternatively, the loss in dlPFC alpha activities may be associated with a decrease in motor preparation, which led to increased SRTs. Given the stronger increase in the SRTs of antisaccades compared with prosaccades, such a preparatory function must also have a cognitive control component, which is consistent with the role proposed for both the dlPFC and the dACC in the extended saccade network [1].

Tasks demanding cognitive control engage a network involving multiple brain regions, each of which partakes in this process through oscillatory and spiking activities occurring at coordinated timing, e.g., through phase synchronization or cross-frequency coupling [83–85]. This theory implies a pitfall in behavioral analysis: when dACC is deactivated, a function supported by the dlPFC may appear impaired due to the absence of a dACC signal that communicates the need. Without the simultaneous recording of activities from the dlPFC during the deactivation, the role of the dlPFC in the behavioral impairment could easily be underestimated and the role of dACC overestimated. Furthermore, our discussion of the roles of dACC and dlPFC may still contain some overestimation, since we did not simultaneously record neural activities from other regions in the extended cognitive control network, e.g., the posterior parietal cortex. Therefore, future studies combining electrophysiological recording from more than 1 brain region with reversible deactivation will likely generate new and more precise knowledge on the functional roles of both individual regions and their communication during cognitive processes.

In summary, using an uncued rule-switching task, we found that dACC deactivation impaired performance maintenance after shifting to the new rule rather than disrupting the rule switch per se. While our findings do not preclude the role of the dACC in error monitoring and feedback-related processing, they strongly support its function in maintaining optimal performance, especially in a task with greater cognitive demands [25–31,86]. Importantly, our finding indicates that the dACC may engage cognitive resource partly through its critical role on the task-related theta and performance-related beta activities in the dlPFC. Additionally, the dACC may help motivate effective and prompt responses by enhancing task-related alpha activity in the dlPFC [75]. Taken together, our findings suggest that maintenance of a cognitively challenging rule requires the concerted effort of an extended network including both the dACC and the dlPFC, in which task-relevant oscillations in one region are enabled or facilitated by another.

## Materials and methods

### Ethics statement

All procedures were conducted in accordance with the Canadian Council on Animal Care Policy on the Use of Laboratory Animals and protocol 2008–0125 approved by the Animal Care Committee of the University of Western Ontario Council on Animal Care.

Two adult male macaque monkeys C (*Macaca mulatta*) and T (*M*. *fascicularis*), weighing 6.5 and 9.5 kg, respectively, were used in the study. After the initial chair training, they were implanted with a plastic head restraint for head fixation during training. Once recovered, they were trained on the uncued rule-switch task. A second surgery was then conducted to implant the cryoloops and the microelectrode array once they fully acquired the task. After recovery, they were retrained before testing started.

### Surgical procedures

Each surgery was performed aseptically, with the animals’ physiological parameters continuously monitored and frequently recorded by an experienced veterinary technician. Following each surgery, the animals received analgesics and antibiotics and were monitored by a university veterinarian. In the first surgery, the monkeys were implanted with a plastic head restraint, secured to the skull using bone screws and dental acrylic using previously described aseptic surgical procedures [87]. Upon recovery from the surgery, they were trained to perform the uncued prosaccade and antisaccade switch tasks. Once trained, they underwent a second surgery in which stainless steel cryoloops (8–10 × 3 mm) were implanted bilaterally into the anterior cingulate sulci. The posterior ends of the cryoloops were placed at the same anterior-posterior coordinates as the posterior ends of the principal sulci, such that the cryoloops targeted the dACC (area 24c). Neurons with task-selective activity for prosaccades and antisaccades have been found in this area of the dACC [15]. The technical details of the cryoloop surgery and deactivation method have been previously described [59,64].

In the same surgery, each of the animals was implanted with a 96-channel Utah microelectrode array (Blackrock Microsystems LLC, Salt Lake City, UT). The initial craniotomy for cryoloop placement was extended using rongeurs until the arcuate and principal sulci could be visually identified. The meninges were carefully removed, and the array was placed at the center of area 9/46d in the left hemisphere and inserted with a pneumatically actuated impulse inserter (Blackrock Microsystems LLC, Salt Lake City, UT). A layer of Gel foam was then placed over the exposed brain tissue and dura mater and covered with silicone sealant before dental acrylic was applied to seal the craniotomy. The reference wires were secured underneath the skull and above dura mater. The array was connected by a bundle of fine wires to Omnetics connectors (Omnetics Connector Corporation, Fridley, MN), which were then secured to the surface of the skull with dental cement at a location posterior to the implantation site and were protected by a PEEK chamber covered with a cap. The wire bundle was also secured and covered with dental acrylic.

### Behavioral task

Monkeys were trained to perform and switch between uncued prosaccades and antisaccades (Fig 1C). Each trial began with the presentation of central white fixation spot. Monkeys were required to fixate within a 0.5° × 0.5° window surrounding this spot for a duration of 1.1 s to 1.4 s at the beginning of each trial. Subsequently, the fixation spot was extinguished, and a peripheral white stimulus (0.15°) was presented pseudorandomly with equal probability at an eccentricity of 8° to the left or to the right. To receive a liquid reward, monkeys were required to generate a saccade within 500 ms toward the stimulus on prosaccade trials and away from the stimulus on antisaccade trials, within a 5°× 5° window. After 15–25 correct trials, the task rule switched from prosaccades to antisaccades or vice versa without any explicit signal to the subjects. Consequently, rule switches were guided by trial and error based on the presence or absence of reward after each trial. Several task rule switches were completed within each session.

### Reversible cryogenic deactivation and data acquisition

To deactivate the dACC, chilled methanol was pumped through a cryoloop with Teflon tubing, which passed through a methanol and dry ice (approximately −80°C) bath. Methanol that had passed through the cryoloop was returned to the same reservoir from which it came. Evoked neural activity in underlying cortical tissue is absent when cortical tissue is cooled to below 20°C but returns to normal above 24°C [57,59,88].

Cryoloop temperature was monitored by an attached microthermocouple and maintained at 1–3°C to deactivate as large an area of cortical tissue as possible while avoiding potentially damaging subzero temperatures at the cortical surface [64]. For Monkey T, we had to maintain the cryoloop temperature substantially higher at 14–15°C because the monkey stopped performing the task at lower temperatures. Although for both monkeys we implanted the cryoloops precisely based on sulcal landmarks, the small variation in the amount of cortical tissue contacted by the loops could not be completely avoided. In Monkey T, the cryoloops were likely in direct contact with the cortical tissue, whereas in Monkey C there may be a tiny gap between the cryoloops and tissue surface. Given that tissue temperature rises by 10°C–20°C/mm away from the cryoloops [56], a 0.5-mm difference could account for the variation between subjects. Thus, in Monkey T, cooling to 1°C–3°C would affect a bigger tissue volume than in Monkey C, and the target of 14°C–15°C was probably more appropriate. Previous studies have demonstrated that the impact on neural activity from cooling extends to a maximal 2 mm, and the effect on tissue temperature extends up to 3 mm [59,60]. Each cryoloop measured 8–10 × 3 × 2 mm, thus the volume of deactivated cortex was expected to be within a volume with dimensions 12–14 x 7 x 6 mm, or 504–588 mm^3^; and the volume of cortex that may had a change in temperature was estimated to be within a volume of 14–16 × 9 × 8, or 1,008–1152 mm^3^. Hence, cooling of the cryoloops affected the dorsal and ventral banks of the anterior cingulate sulci, corresponding to area 24c (Fig 1A). Since the implanted array in the dlPFC was approximately 6 mm away from the dACC, it was not affected directly by the reduction in temperature. Additionally, because cooling deactivates neural activity by blocking synaptic transmission [61,62], it would not be expected to affect fibers of passage [64]. Details of the cryoloop procedure have been described previously [59,64].

In each cooling session, the animals performed the task for 30 min without cooling—this baseline epoch followed by a 30-min cooling epoch allowed for the collection of sufficient behavioral and neural data under both conditions. At the end of the 30th minute, the pumps were turned on to start cooling. The first 4 min after the onset of the pumps were excluded from all data analysis to ensure that the cortical tissue adjacent to the cryoloops was cooled below 20°C and that neurons were deactivated (Fig 1B). In addition, both monkeys performed sham sessions that also consisted of 2 consecutive 30-min epochs to control for the effects of time and fatigue over the course of a session. During the second 30-min “control epoch,” the pumps were turned on, but no methanol ran through the cryoloops, and cortical tissue remained active (Fig 1B). Monkey C completed 17 cooling and 17 sham sessions, while Monkey T performed 13 cooling and 14 sham sessions.

Throughout each cooling or sham session, eye movements were recorded at 1,000 Hz with high-speed infrared video eye tracking (Eyelink 1000, Kanata, Ontario, Canada), and the timing of behavioral events were controlled and recorded by the Cortex real-time behavioral control and data acquisition system (NIMH, Bethesda, MD). Both eye tracking and behavioral event timestamps were also sent to and recorded by a Plexon Multichannel Acquisition Processor (MAP) system (Plexon Inc., Dallas, TX), which acquired LFPs and spike trains at 1 kHz from the Utah array. The MAP system synchronizes and combines different types of data into a single file.

### Data analysis

#### Behavioral performance

All analyses were performed using custom Matlab (Mathworks, Natick, MA) code. Saccade onset was identified as the time at which saccade velocity exceeded 30°/s, and saccade offset was identified as the time at which saccade velocity fell below 30°/s. SRT was defined as the time from stimulus onset to saccade onset. Trials with no fixation, broken fixation prior to peripheral stimulus onset, or with SRTs below 80 ms or above 500 ms were excluded from further analyses. Included in the analyses were the direction errors, in which a prosaccade is erroneously made in place of an antisaccade or vice versa.

The initial analysis included 15 pre-switch and 15 post-switch trials from each trial block containing a pro-to-anti or anti-to-pro rule switch (Fig 2). In later analyses, the post-switch trials were broken down to “Early stage,” which includes the 4 trials following the change in task rule, and “Stable stage,” which includes the 5th to 12th trials following the rule switch. Comparing the 2 stages provided insight into how the animals’ behavior progressed from the initial rule change—signaled by omission of reinforcement—to the establishment of the new rule.

#### Signed rule-switch probability

To define post-switch stages, we calculated the signed rule-switch probability. Each prosaccade trial was coded as 1 and antisaccade trial as 0, and then the signed rule switch was calculated by subtracting the code of the previous trial from that of the current one. For instance, the signed rule switch was 1 for a prosaccade trial if it followed an antisaccade trial; it would be −1 for an antisaccade following a prosaccade trial. If a trial followed the same rule as the trial before, then the signed rule switch would be 0. At each post-switch serial position (e.g., first or second trial post switch), we averaged the signed rule switch across all trial blocks to obtain the signed rule-switch probability (Fig 3).

#### Preprocessing and power spectra

LFP data were analyzed in MATLAB (MathWorks, Naticks, MA) using the FieldTrip toolbox (http://fieldtrip.fcdonders.nl/) developed at the Donders Institute for Brain, Cognition and Behaviour [89]. The recorded LFPs were low-pass filtered at 150 Hz, and line noise was removed at 60 Hz and 120 Hz using a discrete Fourier transform. Given that our paper focuses on frequencies above 4 Hz, to remove the electrical noise (approximately 1.2 Hz) and its strongest harmonics generated by the operation of the pumps used to run methanol through the cryoloops, we also high-pass filtered at 3.8 Hz (with no effect at 4 Hz or above). Additional harmonics of the pump noise were removed using the *chunkwiseDeline* function (https://xcorr.net/2011/10/03/removing-line-noise-from-lfps-wideband-signals/) by Patrick Minealt. This function is suitable for mechanical noise with a fixed shape in the time domain. It detects and describes the shape of the noise in the time domain with a family of exponential functions and then subtracts it from the signal. The chunkwise delining method is preferable to the use of a notch filter because it preserves the physiological signals that may occur at the same frequency as the noise. S1 Fig shows an example of this procedure. The high-pass filter at 3.8 Hz removed the primary noise (S1A Fig, top versus middle trace), and the chunk-wise deline procedure removed the higher harmonics (middle versus bottom trace), resulting in a sample (bottom trace) that closely resembles a trace from the baseline epoch of the same channel and session (S1B Fig). From the power spectra averaged across all channels recorded in the session, it was clear that all higher harmonics were thoroughly removed without affecting physiological data (S1C and S1D Fig). The very small remaining artifact component at 4.8 Hz affected less than 0.33% of a 1-Hz window, or 0.082% of the theta band (4–8 Hz). This example does not represent the best of our preprocessing outcome; instead, it was taken from the first session of the animal with more prominent pump-related noise.

The continuous signals were then divided into discrete trials based on event timestamps. The first 1.1 s from all trials including a saccade were included in subsequent analyses, and the 1.1 s preceding the acquisition of fixation in each trial was used as ITI for normalization of LFP power. For the time-frequency presentation of LFP power, the data were processed using a multitaper method with a discrete prolate spheroidal sequence (DPSS) taper set, using a 0.667 s window in time and a 4.5 Hz window in frequency for power spectra. To compare LFP between channels and animals and to reduce variability, we used decibel normalization for each channel at each frequency [90]:

$$P_{Norm}=10\times log_{10}\left(P_{Raw}./P_{ITI}\right)$$

where P_Raw_ refers to the LFP power during the fixation period and P_ITI_ refers to power during ITIs prior to fixation.

#### Experimental design and statistical analyses

The animals’ behavioral performance, calculated as percentage correct in a 15-trial block, was analyzed with repeated-measures ANOVA, with session type (cooling or control) as the categorical factor and epoch (baseline or cooling/control) and switch condition (pre- or post-switch) as repeated measures (Fig 2). The pre-switch performance was calculated from the last 15 trials before a rule switch, and the post-switch performance was calculated from first 15 trials after the switch. The sample size was the number of trial blocks, pooled across sessions, ranging from 152 to 225 depending on the session type, epoch, or switch condition. The SRTs were analyzed similarly, with one averaged SRT used for each trial block (Fig 2). To quantify behaviors from different post-switch stages, because there were only 4 or 8 trials in each stage in a given trial block, we calculated the response accuracy or averaged SRTs based on all trials from that stage from a session, resulting in a sample size equal to session number (*N* = 30 and 31 for cooling and control, respectively; Fig 4).

Because the microelectrodes remained in the same locations in the brain throughout the experiment, for each animal, for the neural analyses we pooled trials recorded from different sessions and computed the power spectra using all trials with a completed response in each rule, epoch, and session type. All neural analyses focused on the fixation period in each trial, which was when the animals had to prepare for the upcoming peripheral stimulus by retaining the current task rule. For statistical comparison between power spectra, we used a nonparametric cluster-based permutation test [91], the sample size being the number of channels (*n* = 96, with 48 from each animal). First, a map of *t*-statistics was calculated between the 2 power spectra (i.e., power values at each time bin and frequency), and clusters were identified using *clusterdata* function in MATLAB. Then, for each cluster we obtained a nonparametric statistic, calculated as the cluster sum of *t*-statistics. The significance level was then determined using a distribution of cluster-summed *t*-statistics generated by 5,000 iterations of the same process as above, after randomly splitting and pooling the original data. The cluster-summed *t-*statistics from the real data that were greater than 99.9% of the generated distribution were considered significant. When multiple comparisons were to be made using this test (Figs 6 and 8), instead of repeating the test 4 times, we tiled together the power spectra from within a session type and tested between the 2 session types. This way, cluster-summed *t-*statistics across all conditions had to pass the criterion determined by a single shuffled distribution rather than multiple different distributions, making the results more comparable across conditions.

## Supporting information

S1 Text Detailed methods on the binomial logistic regression and results from the analysis, which revealed no evidence that the animals adopted incorrect strategies beyond the task rules, with or without dACC deactivation. dACC, dorsal anterior cingulate cortex.
(DOCX)

S1 Fig Example of artifact removal without affecting physiological data. (A) A 4-s original time series of (LFPs during a cooling epoch from a single channel (top trace), high-pass filtered at 3.8 Hz (middle trace), underwent additional artifact removal using the chunk-wise deline method (bottom trace). The bottom trace closely resembles (B), which shows a 4-s original series of LFPs from the baseline period before cooling onset. (C) Fourier spectrum calculated from the cooling epoch of the same session as (A) and (B), averaged across all channels, before (top) and after (bottom) artifact removal. The harmonics became negligible at frequencies greater than 15 Hz. (D) Same spectrum as in (C), zoomed in on the theta range where the artifacts were the strongest. LFP, local field potential.
(TIF)

S2 Fig Cumulative probability distributions of SRTs under both rules in different epochs and types of sessions. In all cases, the SRTs became longer with epoch (from blue to red curves). (A) On antisaccade trials, direction errors were prosaccades (lower panels) with shorter SRTs than correct antisaccades (upper panels). Compared with the baseline epoch (red curves), both correct and error responses had longer SRTs (blue curves) in the control epoch (left panels) as well as during the cooling epoch (right panels). While the errors (prosaccades under the antisaccade rule) had relatively short SRTs compared with correct antisaccades, these were still longer than the SRTs of correct prosaccades (panel B). (B) Under the prosaccade rule, direction errors were antisaccades (lower panels) with longer SRTs than correct prosaccades (upper panels). Compared with the baseline epoch (red curves), both correct and error responses had longer SRTs (blue curves) in the control epoch (left panels) as well as during the cooling epoch (right panels). Data associated with this figure can be found at 10.6084/m9.figshare.8236589. SRT, saccadic reaction time.
(TIF)

S3 Fig Regression coefficients of the effects of various factors on response accuracy and on response direction. Whether a response was correct could not be predicted from (A) the response direction or (B) the response direction of the previous trial, although (C) the accuracy of the previous trial could be used to predict the accuracy of prosaccade trials. The direction of the animals’ response could not be predicted from (D) the response direction, (E) the response accuracy, or (F) their interaction in the previous trial. Gray dashed lines indicate the regression coefficients (“betas”) during the baseline epochs; black dashed lines indicate the betas during the cooling/control epochs. Gray horizontal bars indicate trial points where the factor significantly predicted the dependent variable during the baseline epochs, whereas black bars indicate significance during the cooling/control epochs (*p* < 0.05). Data associated with this figure can be found at 10.6084/m9.figshare.8236589.
(TIF)

S4 Fig Regression coefficients of the effects of interactive factors on response accuracy. Response accuracy at trial *t* could not be predicted from (A) the interaction between response direction at trial *t* and response accuracy at trial *t −* 1, (B) the interaction between response direction at trial *t* and response direction at trial *t − 1*, or (C) the interaction between response accuracy at trial *t– 1* and response direction at trial *t − 1*. This model was built to test the predictive power of the interactions among the factors included in the main analysis (S3A–S3C Fig). These interactive terms could not be included in the main model due to multicollinearity with the main factors. Gray dashed lines indicate the regression coefficients (“betas”) during the baseline epochs; black dashed lines indicate the betas during the cooling/control epochs. Gray horizontal bars indicate trial points where the factor significantly predicted the dependent variable during the baseline epochs, whereas black bars indicate significance during the cooling/control epochs (*p* < 0.05). Data associated with this figure can be found at 10.6084/m9.figshare.8236589.
(TIF)

S5 Fig Task-related LFP power in theta (4–8 Hz), alpha (9–12 Hz), low beta (13–20 Hz), and high beta (21–30 Hz) frequency bands under different rules and post-switch stages during direction error trials. See S8 Table for the complete set of results from the statistical test on LFP power in both correct and error trials. In each frequency band, LFP power during fixation periods was standardized against LFP power during ITIs. Each plot contains averaged task-related LFP power in baseline (left symbols in each panel) and cooling/control epochs (right symbols) in sham (red) and cooling (blue) sessions, at Early (light red/blue) and Stable (dark red/blue) stages and under prosaccade (upper panels) and antisaccade (lower panels) rules. In alpha and both beta bands (second, third, and fourth panels in both rows), dACC cooling had a negative impact on task-related LFP power on error trials very similar to those in correct trials, under both rules and at both stages. In theta band, the cooling-related decrease in power was observed less often, which was also similar to the findings in correct trials. Data associated with this figure can be found at 10.6084/m9.figshare.8236589. dACC, dorsal anterior cingulate cortex; ITI, intertrial interval; LFP, local field potential.
(TIF)

S1 Table Effects of session type, epoch, and switch on response accuracy before and after antisaccade-to-prosaccade (A→P) switches. Results are also illustrated in Fig 2B. In all cases: d.f. = 1, d.f. for errors = 764.
(XLSX)

S2 Table Effects of session type, epoch, and post-switch stage on SRTs before and after antisaccade-to-prosaccade (A→P) switches. Results are also illustrated in Fig 2B. In all cases: d.f. = 1, d.f. for errors = 763. SRT, saccadic reaction time.
(XLSX)

S3 Table Effects of session type, epoch, and post-switch stage on response accuracy. These results are also illustrated in Fig 4A and 4B. In all cases: d.f. = 1, d.f. for errors = 58.
(XLSX)

S4 Table Effects of session type, epoch, and post-switch stage on SRT. These results are also illustrated in Fig 4C and 4D. In Table A, d.f. = 1, d.f. for errors = 58. In Table B, d.f. = 1, d.f. for errors = 56. SRT, saccadic reaction time.
(XLSX)

S5 Table Epoch-related changes in LFP power in different frequency bands (theta, alpha, low beta, and high beta) in session type versus sham sessions. d.f. = 3 for main or interactive effect of frequency, d.f. = 285 for errors associated with effects of frequency; d.f. = 1 for all other effects, d.f. for errors = 95. LFP, local field potential.
(XLSX)

S6 Table Baseline task-related LFP power in different frequency bands (theta, alpha, low beta, and high beta) during correct and error trials in Early and Stable post-switch stages. These results are also illustrated in Fig 5A. d.f. = 3 for main or interactive effect of Frequency, d.f. = 285 for errors associated with effects of Frequency; d.f. = 1 for all other effects, d.f. for errors = 95. LFP, local field potential.
(XLSX)

S7 Table Effects of epoch and session type on task-related LFP power preceding correct responses in different frequency bands (theta, alpha, low beta, and high beta) in Early and Stable post-switch stages. These results are also illustrated in Fig 5B. d.f. = 3 for main or interactive effect of Frequency, d.f. = 285 for errors associated with effects of Frequency; d.f. = 1 for all other effects, d.f. for errors = 95. LFP, local field potential.
(XLSX)

S8 Table Effects of epoch, session type, rule, stage, and performance (correct versus error) on task-related LFP power in different frequency bands (theta, alpha, low beta, and high beta). d.f. = 3 for main or interactive effect of Frequency, d.f. = 285 for errors associated with effects of Frequency; d.f. = 1 for all other effects, d.f. for errors = 95. LFP, local field potential.
(XLSX)

## Abbreviations

ACC: anterior cingulate cortex
C-E: correct-error
dACC: dorsal anterior cingulate cortex
dlPFC: dorsolateral prefrontal cortex
DPSS: discrete prolate spheroidal sequence
EEG: electroencephalogram
ITI: intertrial interval
KS: Kolmogorov-Smirnov
LFP: local field potential
MAP: Multichannel Acquisition Processor
SRT: saccadic reaction time
