# Supplementary material for: Macaque anterior cingulate cortex deactivation impairs performance and alters lateral prefrontal oscillatory activities in a rule-switching task
Source: PLoS Biol. 2019 Jul 11;17(7):e3000045. doi: 10.1371/journal.pbio.3000045 (PMC6650082; doi:10.1371/journal.pbio.3000045)
Supplement: S1 Text — dACC, dorsal anterior cingulate cortex. (DOCX) [file pbio.3000045.s001.docx]

**SUPPLEMENTARY METHODS**

**Binomial logistic regression**

The purpose of this analysis was to systematically examine whether factors *a)* saccadic direction of the previous trial *t – 1*, *b)* saccadic direction of the current trial *t* and *c)* response accuracy on *t – 1* predicted the accuracy and direction of the response on the current trial *t*. Three versions of this analysis were conducted: *1)* factors *a, b* and *c* as predictors, accuracy at trial *t* as the dependent variable; *2)* factors *a, b* and *c* as predictors and response direction at trial *t* as the dependent variable; *3)* the 3 interaction terms between factors *a, b* and *c* as predictors (i.e. *a* × *b*, *a* × *c* and *b* × *c*) and accuracy at trial *t* as the dependent variable. For response accuracy, correct is coded as 1 and error as –1. For response direction, right is coded as 1 and left as –1.

We first pooled trials from all sessions, each labeled with reference to both the previous and to the subsequent rule switch point. For instance, a given prosaccade trial could be labeled as +8 with reference to the previous antisaccade-to-prosaccade (A🡪P) switch point, and –10 with reference to the next prosaccade-to-antisaccade (P🡪A) switch point. The trial would be part of the regression model for A🡪P switches consists of all trials labeled +8, as well as part of a different model for P🡪A switches consists of all trials labeled –10. Then for each trial point before and after a rule switch, we conducted binomial logistic regression using the MATLAB *mnrfit* function with nominal model. We then plotted regression coefficients (‘betas’) with horizontal bars indicating the significance of each beta value (S3 Fig and S4 Fig).

**SUPPLEMENTARY RESULTS**

**Animals Did Not Adopt Incorrect Strategies Beyond the Task Rules with or without dACC Deactivation**

Given the uncued, feedback-based nature of our task, the outcome of the previous trial was expected to influence the animals’ response on the current trial—an influence that was likely to vary depending on the task rule and the number of trials elapsed since rule switch. Additionally, when the animals had difficulty choosing the correct response after a rule switch, it was possible that they temporarily adopted an incorrect strategy of direction, i.e. looking to the right if a previous rightward saccade was rewarded, regardless of the current location of the stimulus. We used a binomial logistic regression model at each trial point before and after a rule switch to systematically examine whether 1) saccadic direction of the previous trial *t – 1*, 2) saccadic direction of the current trial *t* and 3) response accuracy on *t – 1* predicted the outcome of the current trial *t*. We then plotted the regression coefficients (or ‘betas’) from models from trials from –14 to +15 (with trial ‘0’ being the last pre-switch trial), with each model examining 204 or more trials, including at least 95 trials from each animal (S3 Fig, dashed lines, gray for baseline epochs, black for cooling/control epochs). Significant beta values were indicated by solid horizontal bars with the same color. We found that the response direction of either trial *t* or *t* – *1* had little influence on the outcome of the current trial *t*, whether during the baseline (gray dashed lines) or the cooling/control epoch (black dashed lines) in sham sessions (S3 Fig A and B, left columns) or in cooling sessions (right columns) at most pre- or post-switch trial positions. This was the case even when the performance was at the lowest level soon after a rule switch according to Fig 2. By contrast, response accuracy on trial *t* – *1* significantly influenced the outcome of the trial *t* in most of the prosaccade trials (S3 Fig C, lower panels, positive side) and the first few antisaccade trials after a rule switch during the baseline epochs (upper panels, positive side, gray curves and horizontal bars). While this influence stayed the same for post-switch prosaccade trials across epochs and sessions (S3 Fig C, lower panels), it affected less antisaccade trials during the control epoch than the baseline epoch in sham sessions (top left panel, black vs. gray horizontal bar). The same was true when the cooling epoch was compared to the baseline epoch (top right panel, black vs. gray horizontal bar). Hence, this change appeared to be an effect of epoch rather than cooling per se.

While the three factors in the model were mutually independent, introducing interactive terms would lead to correlation among factors (i.e. multicollinearity) and instability in the resulting regression coefficients. Hence, we constructed a separate model consisting solely of the interactive terms which were orthogonal to each other: 1) response direction at *t* × accuracy at *t – 1*, 2) response direction at *t* × response direction at *t – 1* and 3) accuracy at *t – 1* × response direction at *t – 1*. Only when accuracy at *t – 1* (Models 1 and 3) was involved did we detect transient predictive power (S4 Fig A and C, gray and black horizontal bars); the interactive term involving response direction only (Model 2) did not have any predictive power over the accuracy at trial *t* (S4 Fig B). In summary, the animals’ response accuracy was independent of the current response direction, thus side bias could not have contributed to their performance; it was independent of the previous response direction, thus a strategy of choosing one side repeatedly regardless of the target could not have contributed to their performance either.

Interestingly, the animals’ response accuracy could be predicted from accuracy at *t – 1* on most prosaccade trials but only the first few antisaccade trials post switch. On prosaccade trials this positive relationship indicated that correct responses tended to be repeated. On early post-switch antisaccade trials which contained more error responses, the significant and negative beta values indicated that errors were likely to be corrected on subsequent trials—a phenomenon appeared to weaken from baseline to cooling/control epochs. During subsequent trials in the blocks, the animals may rely on a lasting representation of the antisaccade rule rather than making decisions based on the outcome at *t – 1*.

We performed an additional set of logistic regressions to examine the influence of saccadic direction and response accuracy on the *saccadic direction* of the following trial*.* Perhaps the animals tended to incorrectly repeat the same movements when they were unclear with the task rule. This turned out not to be the case: the response direction at *t – 1* was rarely predictive of the response direction at *t* under either rule and during any epoch (S3 Fig D). Nor was the response accuracy at *t – 1* predictive of the current saccadic direction (S3 Fig E). In short, the animals did not adopt a fixed response direction under any of the conditions examined. While cooling impaired their performance (Fig 2), when making a correct antisaccade response, they were likely guided by the task rule rather than any alternative erroneous strategy. In summary, we found no sign that the animals adopted an incorrect strategy (e.g. fixed response direction) beyond the task rules, with or without dACC deactivation.
