# Supplementary figures and images for: Macaque anterior cingulate cortex deactivation impairs performance and alters lateral prefrontal oscillatory activities in a rule-switching task
Source: PLoS Biol. 2019 Jul 11;17(7):e3000045. doi: 10.1371/journal.pbio.3000045 (PMC6650082; doi:10.1371/journal.pbio.3000045)

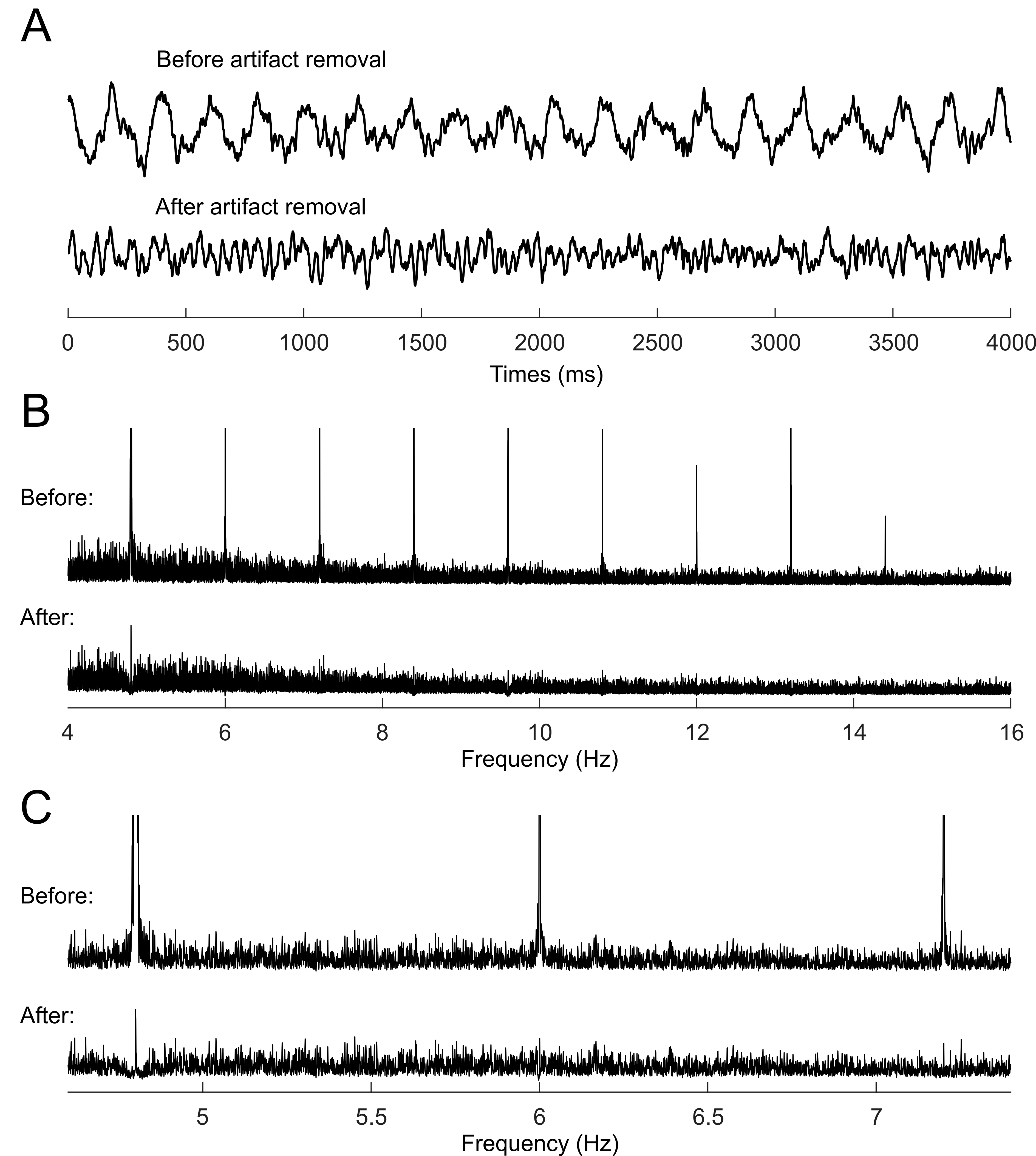

Supplement: S1 Fig — (A) A 4-s original time series of (LFPs during a cooling epoch from a single channel (top trace), high-pass filtered at 3.8 Hz (middle trace), underwent additional artifact removal using the chunk-wise deline method (bottom trace). The bottom trace closely resembles (B), which shows a 4-s original series of LFPs from the baseline period before cooling onset. (C) Fourier spectrum calculated from the cooling epoch of the same session as (A) and (B), averaged across all channels, before (top) and after (bottom) artifact removal. The harmonics became negligible at frequencies greater than 15 Hz. (D) Same spectrum as in (C), zoomed in on the theta range where the artifacts were the strongest. LFP, local field potential. (TIF) [file pbio.3000045.s002.tif]

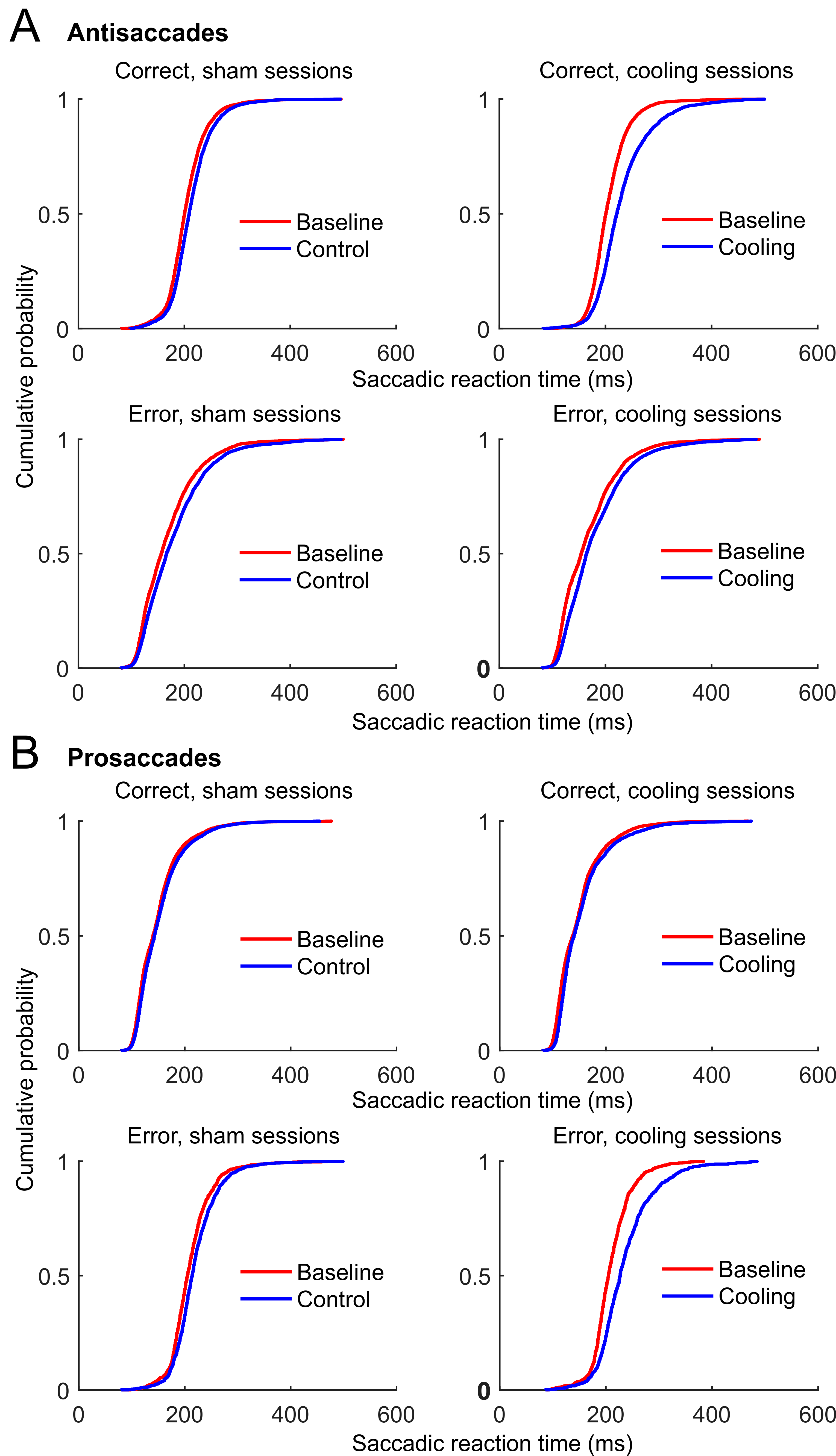

Supplement: S2 Fig — In all cases, the SRTs became longer with epoch (from blue to red curves). (A) On antisaccade trials, direction errors were prosaccades (lower panels) with shorter SRTs than correct antisaccades (upper panels). Compared with the baseline epoch (red curves), both correct and error responses had longer SRTs (blue curves) in the control epoch (left panels) as well as during the cooling epoch (right panels). While the errors (prosaccades under the antisaccade rule) had relatively short SRTs compared with correct antisaccades, these were still longer than the SRTs of correct prosaccades (panel B). (B) Under the prosaccade rule, direction errors were antisaccades (lower panels) with longer SRTs than correct prosaccades (upper panels). Compared with the baseline epoch (red curves), both correct and error responses had longer SRTs (blue curves) in the control epoch (left panels) as well as during the cooling epoch (right panels). Data associated with this figure can be found at 10.6084/m9.figshare.8236589. SRT, saccadic reaction time. (TIF) [file pbio.3000045.s003.tif]

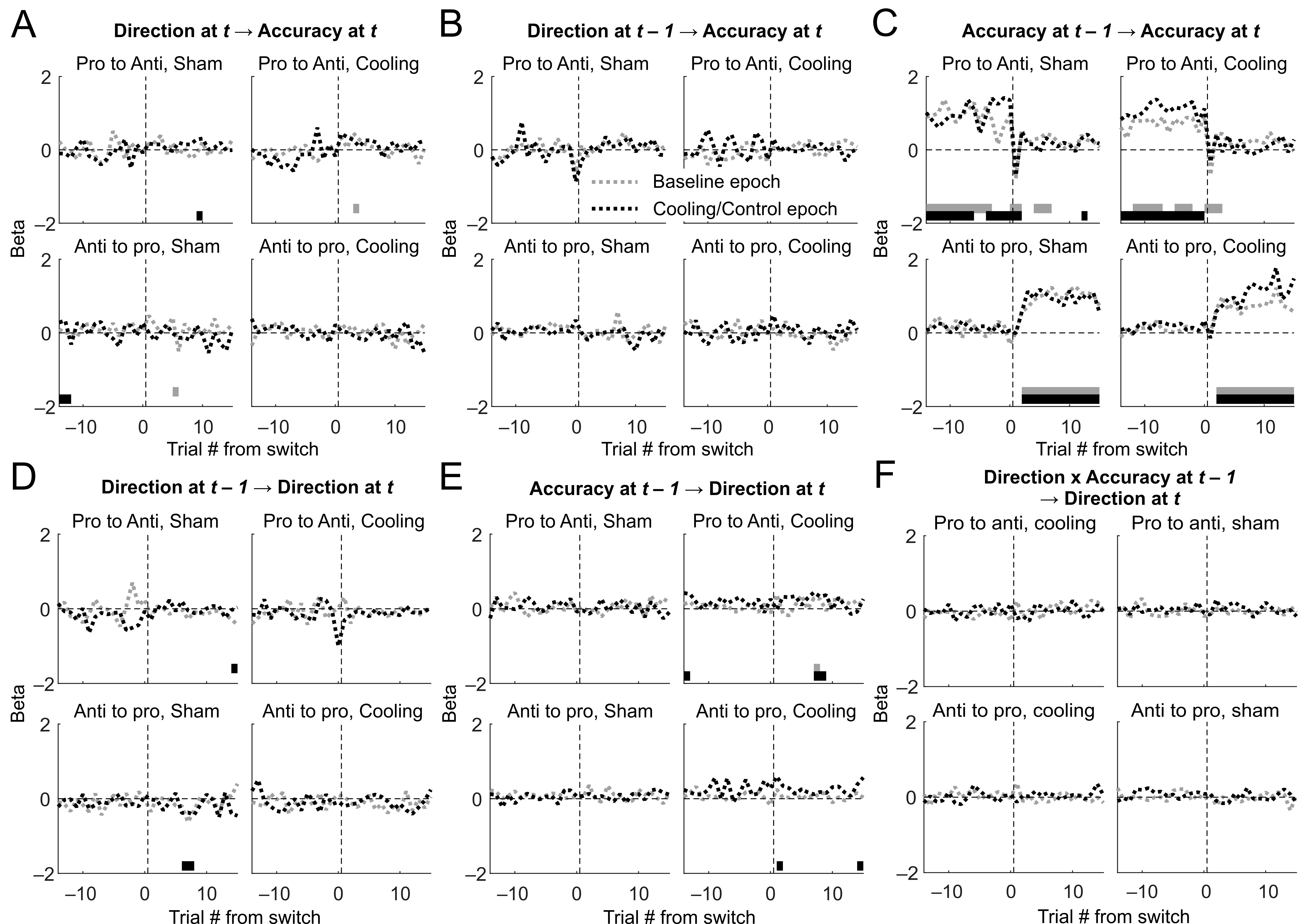

Supplement: S3 Fig — Whether a response was correct could not be predicted from (A) the response direction or (B) the response direction of the previous trial, although (C) the accuracy of the previous trial could be used to predict the accuracy of prosaccade trials. The direction of the animals’ response could not be predicted from (D) the response direction, (E) the response accuracy, or (F) their interaction in the previous trial. Gray dashed lines indicate the regression coefficients (“betas”) during the baseline epochs; black dashed lines indicate the betas during the cooling/control epochs. Gray horizontal bars indicate trial points where the factor significantly predicted the dependent variable during the baseline epochs, whereas black bars indicate significance during the cooling/control epochs (p < 0.05). Data associated with this figure can be found at 10.6084/m9.figshare.8236589. (TIF) [file pbio.3000045.s004.tif]

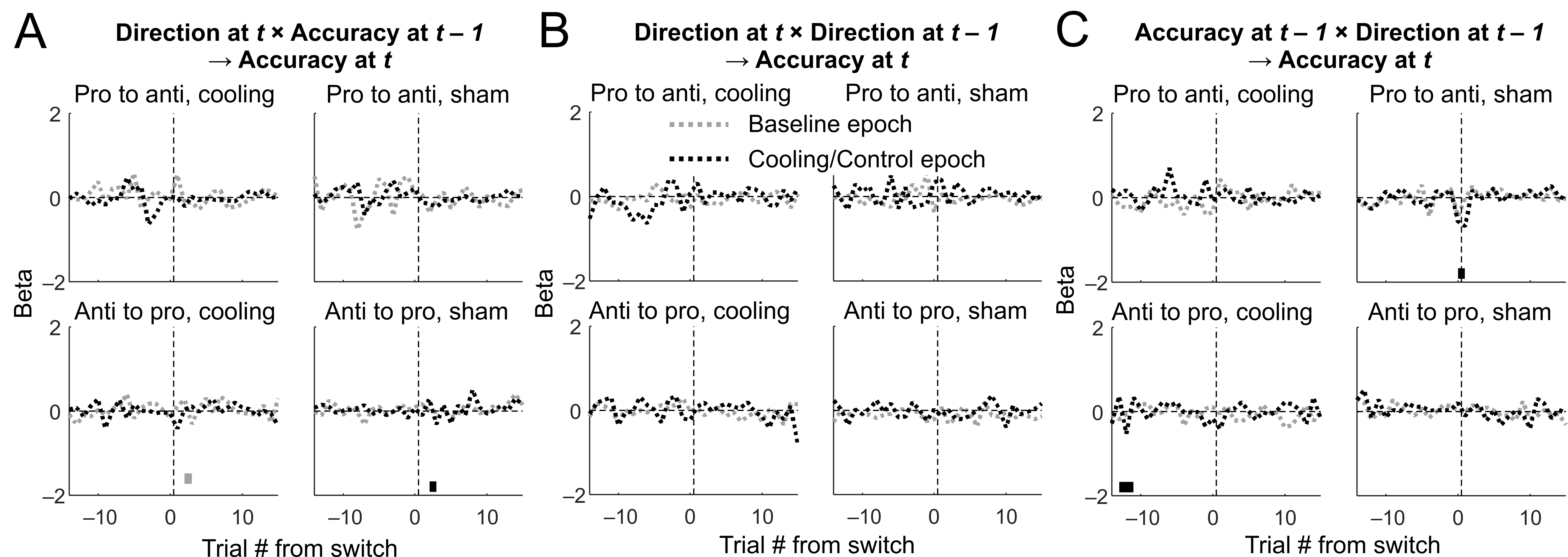

Supplement: S4 Fig — Response accuracy at trial t could not be predicted from (A) the interaction between response direction at trial t and response accuracy at trial t − 1, (B) the interaction between response direction at trial t and response direction at trial t − 1, or (C) the interaction between response accuracy at trial t– 1 and response direction at trial t − 1. This model was built to test the predictive power of the interactions among the factors included in the main analysis (S3A–S3C Fig). These interactive terms could not be included in the main model due to multicollinearity with the main factors. Gray dashed lines indicate the regression coefficients (“betas”) during the baseline epochs; black dashed lines indicate the betas during the cooling/control epochs. Gray horizontal bars indicate trial points where the factor significantly predicted the dependent variable during the baseline epochs, whereas black bars indicate significance during the cooling/control epochs (p < 0.05). Data associated with this figure can be found at 10.6084/m9.figshare.8236589. (TIF) [file pbio.3000045.s005.tif]

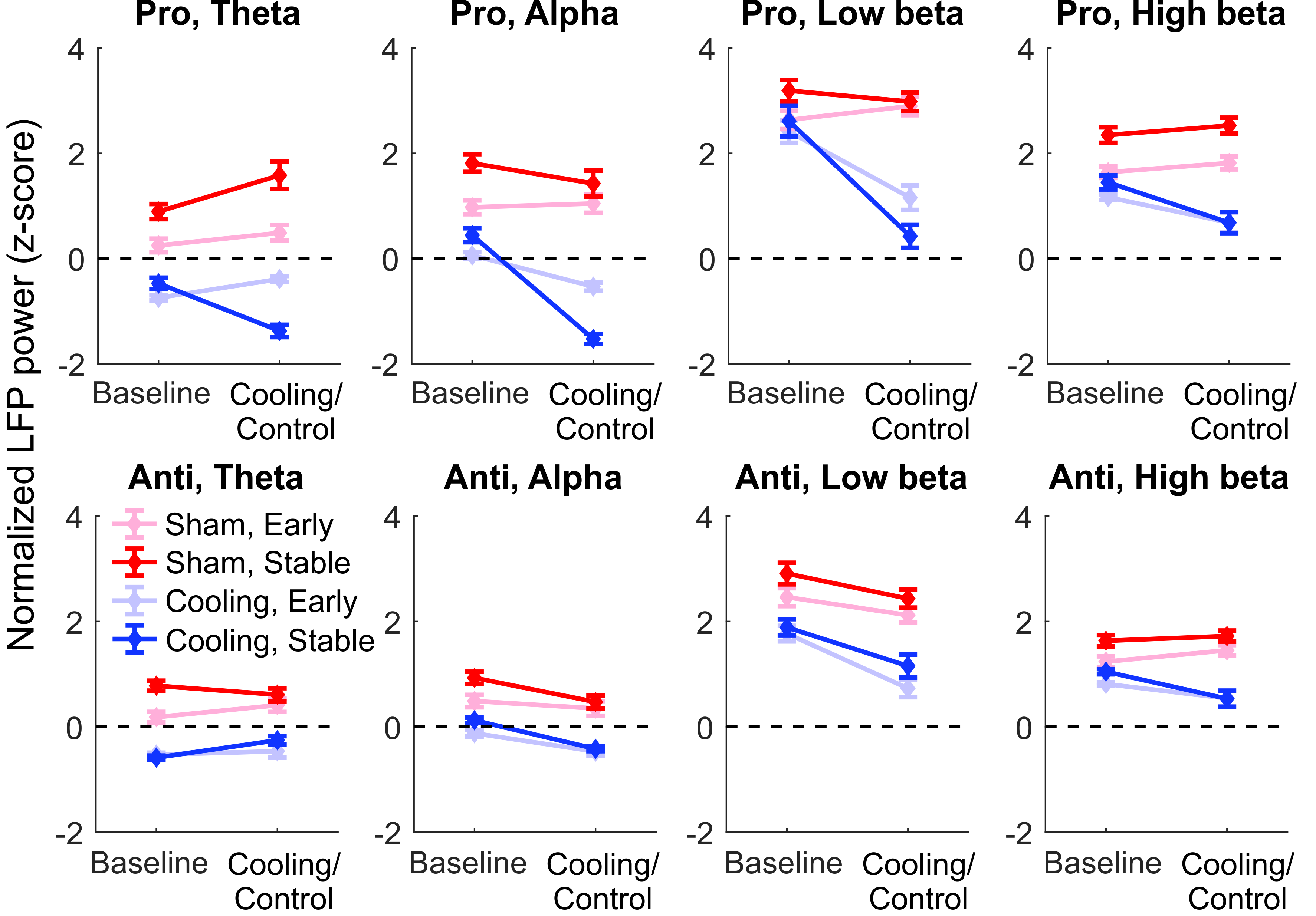

Supplement: S5 Fig — See S8 Table for the complete set of results from the statistical test on LFP power in both correct and error trials. In each frequency band, LFP power during fixation periods was standardized against LFP power during ITIs. Each plot contains averaged task-related LFP power in baseline (left symbols in each panel) and cooling/control epochs (right symbols) in sham (red) and cooling (blue) sessions, at Early (light red/blue) and Stable (dark red/blue) stages and under prosaccade (upper panels) and antisaccade (lower panels) rules. In alpha and both beta bands (second, third, and fourth panels in both rows), dACC cooling had a negative impact on task-related LFP power on error trials very similar to those in correct trials, under both rules and at both stages. In theta band, the cooling-related decrease in power was observed less often, which was also similar to the findings in correct trials. Data associated with this figure can be found at 10.6084/m9.figshare.8236589. dACC, dorsal anterior cingulate cortex; ITI, intertrial interval; LFP, local field potential. (TIF) [file pbio.3000045.s006.tif]
